# Supplementary material for: A systems biology analysis of lipolysis and fatty acid release from adipocytes in vitro and from adipose tissue in vivo
Source: PLoS One. 2021 Dec 31;16(12):e0261681. doi: 10.1371/journal.pone.0261681 (PMC8719686; doi:10.1371/journal.pone.0261681)
Supplement: S1 Table — The parameters were allowed to vary in the range given in S2 Table. For the specific parameter being investigated, the bound was relaxed and the threshold for when a parameter was deemed nonidentifiable was set to the value given in the table in columns Lower threshold and Upper threshold). The minimum and maximal found values of a parameter is given in columns θoriginalmin, and θoriginalmax respectively. (PDF) [file pone.0261681.s001.pdf]

Parameter	Lower threshold	Upper threshold	$\theta^{min}_{original}$	$\theta^{max}_{original}$
$kdrift$	10^{-9}	10^{9}	$3.4443 \cdot 10^{-5}$	$9.1549 \cdot 10^{3}$
$k1a$	10^{-9}	10^{9}	$1.5021 \cdot 10^{-7}$	$6.4646 \cdot 10^{1}$
$k1a2$	10^{-9}	10^{9}	$4.6632 \cdot 10^{-5}$	1.6736
$k1b$	10^{-9}	10^{9}	$1.5857 \cdot 10^{-2}$	$4.2019 \cdot 10^{2}$
$k2a$	10^{-9}	10^{9}	$9.6332 \cdot 10^{-6}$	$1.2044 \cdot 10^{6}$
$k2b$	10^{-9}	10^{9}	$9.1455 \cdot 10^{-4}$	$1.3983 \cdot 10^{6}$
$k3a$	10^{-9}	10^{9}	$4.4438 \cdot 10^{-4}$	$1.0000 \cdot 10^{9}$
$k3a2$	10^{-9}	10^{9}	$2.8713 \cdot 10^{-2}$	$3.4486 \cdot 10^{3}$
$k3b$	10^{-9}	10^{9}	$3.4584 \cdot 10^{-1}$	$9.0966 \cdot 10^{4}$
$k4a$	10^{-9}	10^{9}	$7.3391 \cdot 10^{-4}$	$3.0223 \cdot 10^{2}$
$k4a2$	10^{-9}	10^{9}	$2.1891 \cdot 10^{-6}$	2.2772
$k4b$	10^{-9}	10^{9}	$2.6237 \cdot 10^{-1}$	$5.6350 \cdot 10^{5}$
$k5a$	10^{-9}	10^{9}	$7.4564 \cdot 10^{-2}$	$1.6026 \cdot 10^{5}$
$k5b$	10^{-9}	10^{9}	$1.4359 \cdot 10^{-2}$	$4.2477 \cdot 10^{3}$
$k6a$	10^{-9}	10^{9}	$3.2008 \cdot 10^{-4}$	$5.4919 \cdot 10^{5}$
$k6b$	10^{-9}	10^{9}	$4.9210 \cdot 10^{-3}$	$7.2023 \cdot 10^{5}$
$k7a$	10^{-9}	10^{9}	$2.0387 \cdot 10^{-9}$	$7.5539 \cdot 10^{3}$
$k7b$	10^{-9}	10^{9}	$3.5305 \cdot 10^{-2}$	$5.4970 \cdot 10^{5}$
$k8a$	10^{-9}	10^{9}	$1.7294 \cdot 10^{-4}$	$1.6436 \cdot 10^{4}$
$k8b$	10^{-9}	10^{9}	$3.0972 \cdot 10^{-2}$	1.1554
$k8c$	10^{-9}	10^{9}	$1.0000 \cdot 10^{-9}$	$1.8558 \cdot 10^{-2}$
phe_effect	0	1	0.0000	1.0000
$isoscale$	0	10^{2}	4.2267	$1.0000 \cdot 10^{2}$
min_1	0	10^{2}	$1.5179 \cdot 10^{-1}$	$1.2681 \cdot 10^{1}$
min_2	0	10^{2}	$1.4806 \cdot 10^{1}$	$3.0239 \cdot 10^{1}$
min_3	0	10^{2}	0.0000	$2.0000 \cdot 10^{1}$
$EC50_1$	10^{-6}	10^{4}	$4.3451 \cdot 10^{-2}$	$1.2152 \cdot 10^{1}$
$EC50_2$	10^{-6}	10^{4}	2.3564	$3.6085 \cdot 10^{1}$
$EC50_3$	10^{-6}	10^{4}	$1.0000 \cdot 10^{-6}$	$1.0000 \cdot 10^{4}$
n_1	0	4	$3.8330 \cdot 10^{-1}$	1.1091
n_2	0	4	$5.6093 \cdot 10^{-1}$	4.0000
n_3	0	4	0.0000	4.0000
$diab$	—	—	—	—

All bounds and estimated values for the free parameters. The parameters were allowed to vary in the range given in S2 Table. For the specific parameter being investigated, the bound was relaxed and the threshold for when a parameter was deemed nonidentifiable was set to the value given in the table in columns Lower threshold and Upper threshold). The minimum and maximal found values of a parameter is given in columns $\theta^{min}_{original}$, and $\theta^{max}_{original}$ respectively.
