# Supplementary material for: A systems biology analysis of lipolysis and fatty acid release from adipocytes in vitro and from adipose tissue in vivo
Source: PLoS One. 2021 Dec 31;16(12):e0261681. doi: 10.1371/journal.pone.0261681 (PMC8719686; doi:10.1371/journal.pone.0261681)
Supplement: S2 Table — The rate parameters (kx) were given a free range (10−6 to 106). isoscale was allowed a 20% deviation from the expected value of 10. For the input functions, the minimum values minx was given a range from zero to 20% of max, the steepness nx was given a range from 0 to 2, and the EC50x was given a free range for all doses used in the dataset from [15] (10−5 to 103 nM), except for EC501 which was limited based on the EC50 of IR in [17]. θoriginal* corresponds to the optimal parameter set for the original dataset. θextended* corresponds to the optimal parameter set for the extended dataset. (PDF) [file pone.0261681.s002.pdf]

Parameter	Lower bound	Upper bound	$\theta^*_{original}$	$\theta^*_{extended}$
$kdrift$	10^{-6}	10^{6}	1.8649	2.6525
$k1a$	10^{-6}	10^{6}	$2.5929 \cdot 10^{-2}$	$8.0752 \cdot 10^{-2}$
$k1a2$	10^{-6}	10^{6}	$2.7345 \cdot 10^{-2}$	$1.2840 \cdot 10^{-2}$
$k1b$	10^{-6}	10^{6}	$6.1169 \cdot 10^{-1}$	$2.0825 \cdot 10^{-1}$
$k2a$	10^{-6}	10^{6}	6.5912	$1.2136 \cdot 10^{-2}$
$k2b$	10^{-6}	10^{6}	$2.0591 \cdot 10^{1}$	$9.4937 \cdot 10^{-3}$
$k3a$	10^{-6}	10^{6}	9.4628	$3.0140 \cdot 10^{1}$
$k3a2$	10^{-6}	10^{6}	$1.1379 \cdot 10^{1}$	$1.4944 \cdot 10^{1}$
$k3b$	10^{-6}	10^{6}	$9.2000 \cdot 10^{1}$	$1.2626 \cdot 10^{2}$
$k4a$	10^{-6}	10^{6}	$8.1360 \cdot 10^{-1}$	1.1020
$k4a2$	10^{-6}	10^{6}	$3.9464 \cdot 10^{-3}$	$1.5750 \cdot 10^{-2}$
$k4b$	10^{-6}	10^{6}	$5.6524 \cdot 10^{2}$	$1.2609 \cdot 10^{2}$
$k5a$	10^{-6}	10^{6}	$2.6695 \cdot 10^{2}$	$2.8775 \cdot 10^{1}$
$k5b$	10^{-6}	10^{6}	3.0725	2.0128
$k6a$	10^{-6}	10^{6}	$4.0644 \cdot 10^{-2}$	$4.1938 \cdot 10^{-1}$
$k6b$	10^{-6}	10^{6}	$8.9551 \cdot 10^{-3}$	$2.4450 \cdot 10^{-1}$
$k7a$	10^{-6}	10^{6}	$2.1389 \cdot 10^{-1}$	$9.7287 \cdot 10^{-2}$
$k7b$	10^{-6}	10^{6}	$5.6389 \cdot 10^{1}$	$1.8381 \cdot 10^{2}$
$k8a$	10^{-6}	10^{6}	$6.5756 \cdot 10^{1}$	$2.6920 \cdot 10^{3}$
$k8b$	10^{-6}	10^{6}	$3.8648 \cdot 10^{-2}$	$3.4005 \cdot 10^{-2}$
$k8c$	10^{-6}	10^{6}	$5.4205 \cdot 10^{-3}$	$2.1333 \cdot 10^{-2}$
phe_effect	0.6	1	$8.8329 \cdot 10^{-1}$	$8.4505 \cdot 10^{-1}$
$isoscale$	8	$1.2 \cdot 10^{1}$	9.4266	8.0000
min_1	0	$2 \cdot 10^{1}$	1.9994	1.1158
min_2	0	$2 \cdot 10^{1}$	$2.00000 \cdot 10^{1}$	$2.0000 \cdot 10^{1}$
min_3	0	$2 \cdot 10^{1}$	0.0000	0.0000
$EC50_1$ (nM)	0.5	1.1	1.2454	1.2454
$EC50_2$ (nM)	10^{-5}	10^{3}	$1.2853 \cdot 10^{1}$	$1.3356 \cdot 10^{1}$
$EC50_3$ (nM)	10^{-5}	10^{3}	2.8643	4.1559
n_1	0.5	2	$6.1281 \cdot 10^{-1}$	$6.6829 \cdot 10^{-1}$
n_2	0.5	2	1.9807	1.7424
n_3	0.5	2	2.0000	2.0000
$diab$	0	1	—	$9.4622 \cdot 10^{-1}$

Bounds used for optimization of the free parameters, and the sets of optimal values. The rate parameters (kx) were given a free range (10^{-6} to 10^{6}). *isoscale* was allowed a 20% deviation from the expected value of 10. For the input functions, the minimum values min_x was given a range from zero to 20% of max, the steepness n_x was given a range from 0 to 2, and the $EC50_x$ was given a free range for all doses used in the dataset from [15] (10^{-5} to 10^{3} nM), except for $EC50_1$ which was limited based on the EC50 of IR in [17]. $\theta^*_{original}$ corresponds to the optimal parameter set for the original dataset. $\theta^*_{extended}$ corresponds to the optimal parameter set for the extended dataset.
