# Supplementary figures and images for: A systems biology analysis of lipolysis and fatty acid release from adipocytes in vitro and from adipose tissue in vivo
Source: PLoS One. 2021 Dec 31;16(12):e0261681. doi: 10.1371/journal.pone.0261681 (PMC8719686; doi:10.1371/journal.pone.0261681)

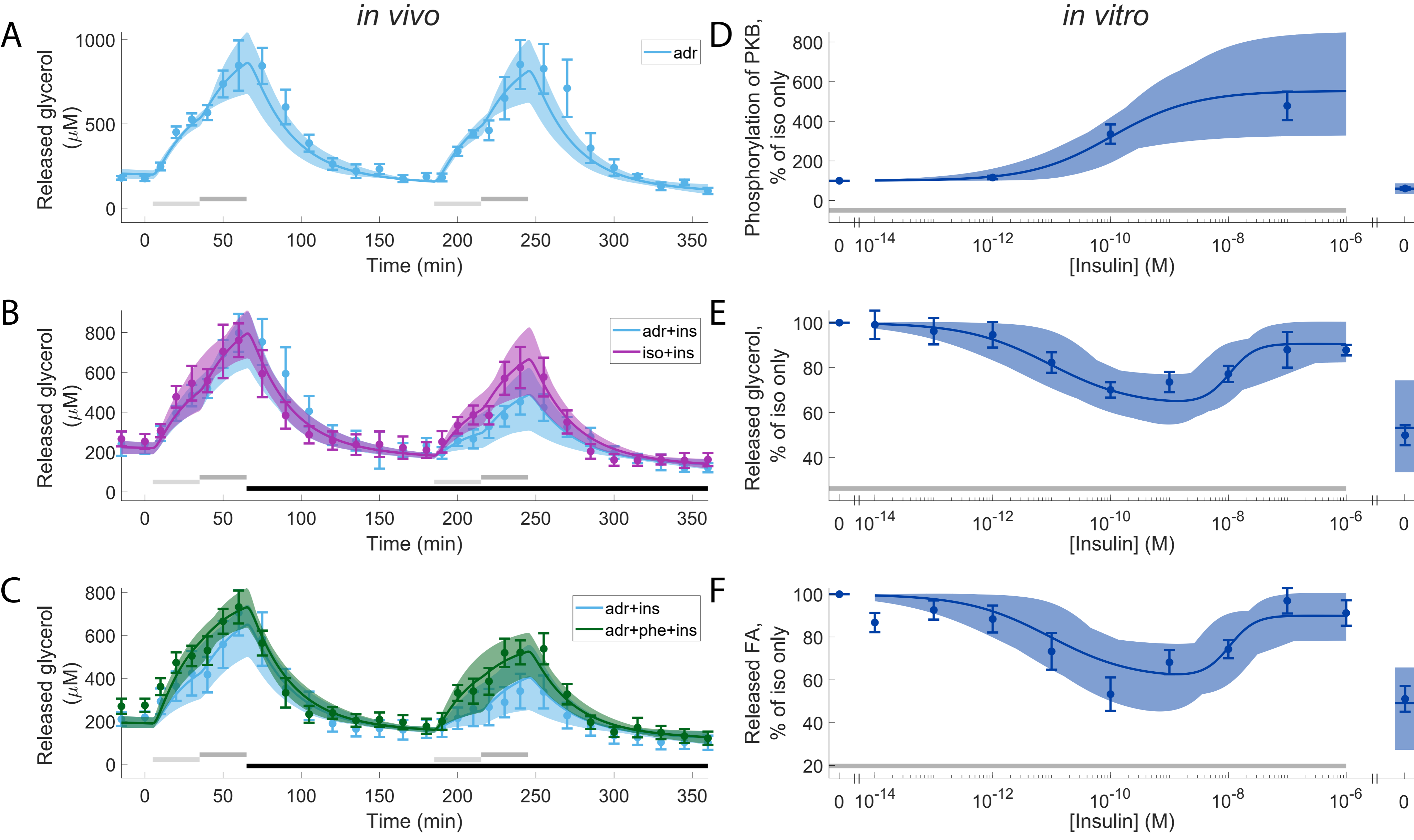

Supplement: S1 Fig — In all panels, solid lines represent the model simulation with the best agreement to data, the shaded areas represent the model uncertainty, and experimental data point are represented as mean values with error bars (SEM). (A-C), in vivo time-series experiments. (D-F), in vitro dose-response experiments. In all subfigures, horizontal bars indicate where stimulations were given. In (A-C), light/dark grey bars indicate low/high adrenergic stimulus (1/10 μM adrenaline or 0.1/1 μM isoproterenol) with or without phentolamine (phe; 100 μM), black bars indicate stimulation with insulin (0.6 nM). In (D-F) grey bars indicate stimulation with isoproterenol (10 nM). In the in vivo experiments, experiments with adrenaline are shown in light blue (A-C), with isoproterenol in purple (B), and with the combined stimulation with adrenaline and phentolamine in green (C). In the in vitro experiments (D-F), increasing doses of insulin were given together with 10nM isoproterenol in all points except one. The point without isoproterenol got no stimulus and is shown to the right in the graphs. (PDF) [file pone.0261681.s003.pdf]

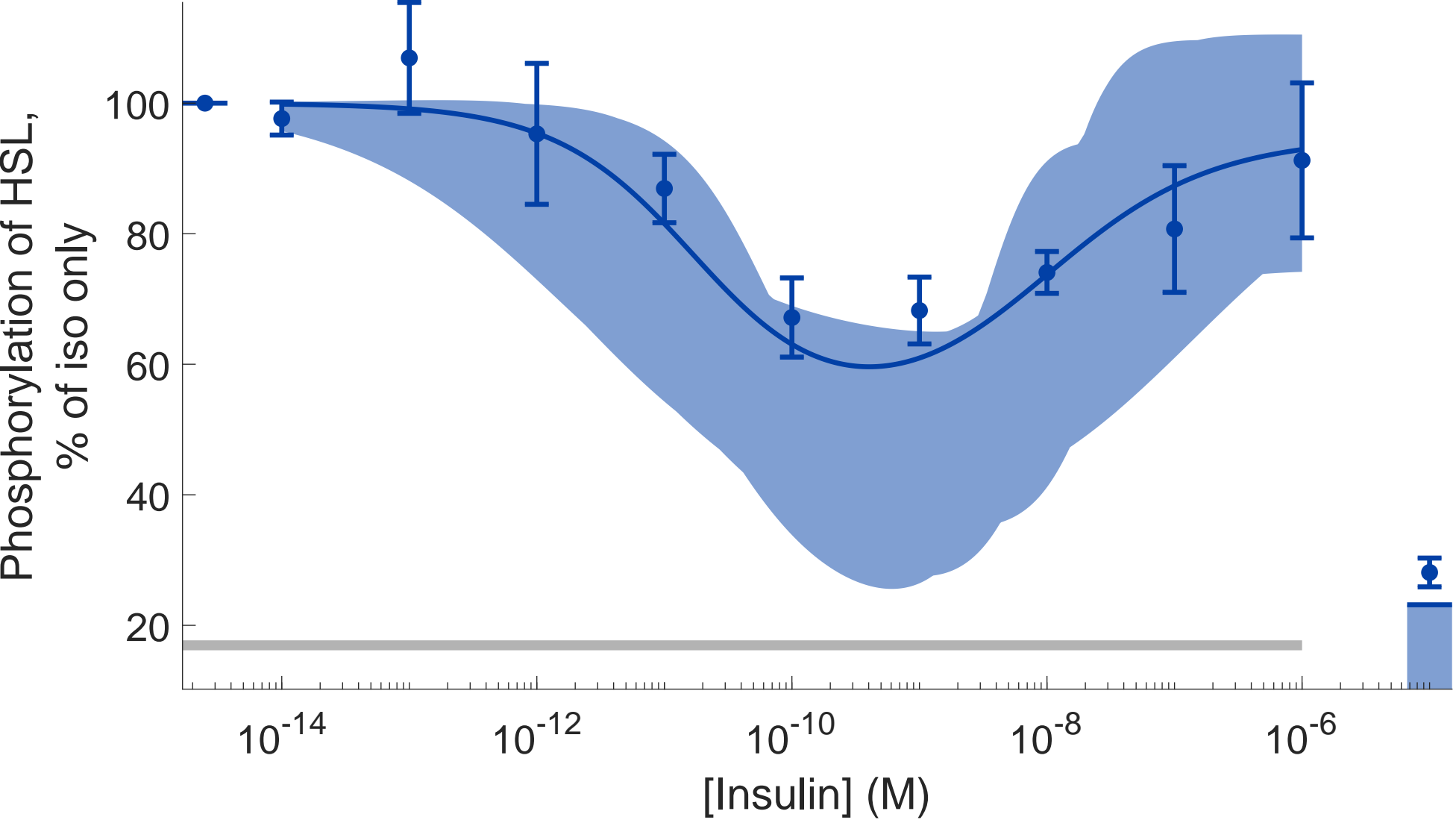

Supplement: S2 Fig — The shaded area represents the model uncertainty, and the experimental data are represented as mean values with error bars (SEM). The horizontal grey bar indicates where stimulation with isoproterenol (10 nM) have been given. Increasing doses of insulin were given together with 10 nM isoproterenol in all points except one. The point without isoproterenol got no stimulus and is shown to the right in the graph. (PDF) [file pone.0261681.s004.pdf]

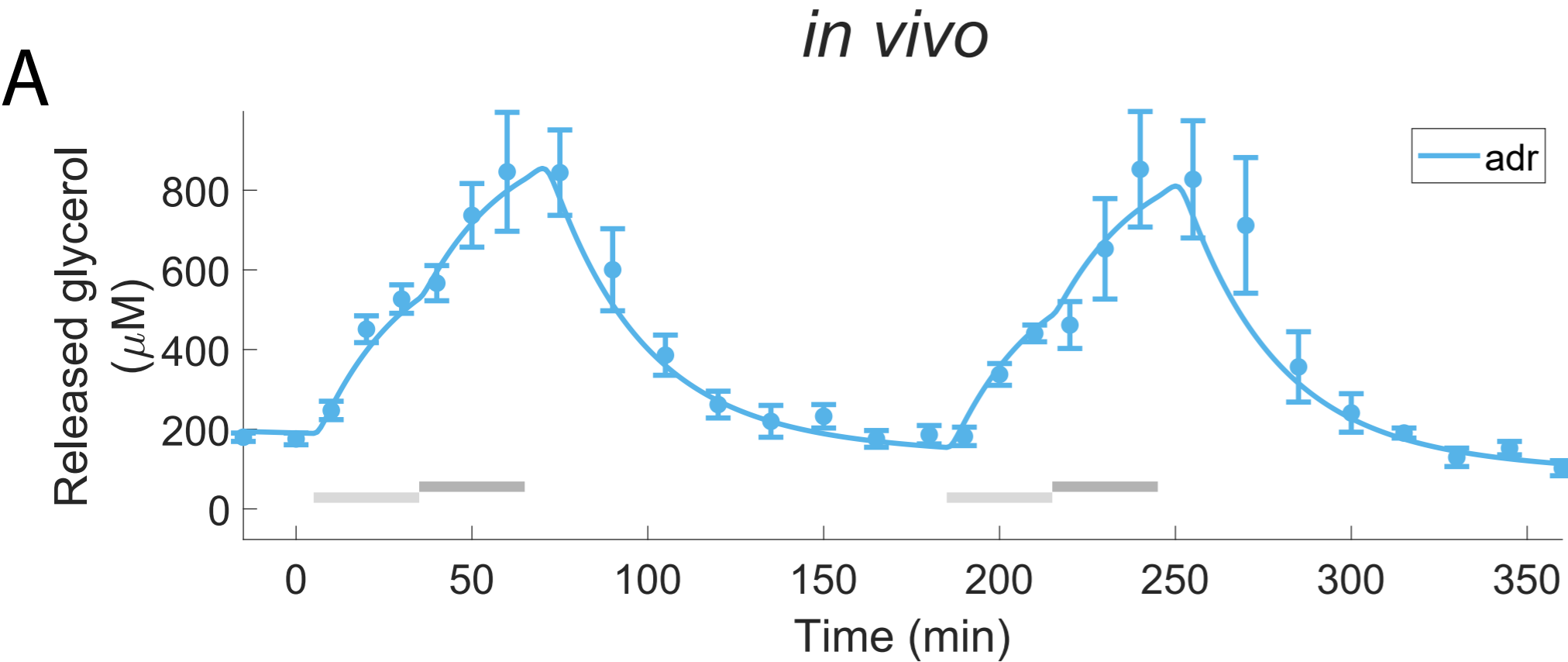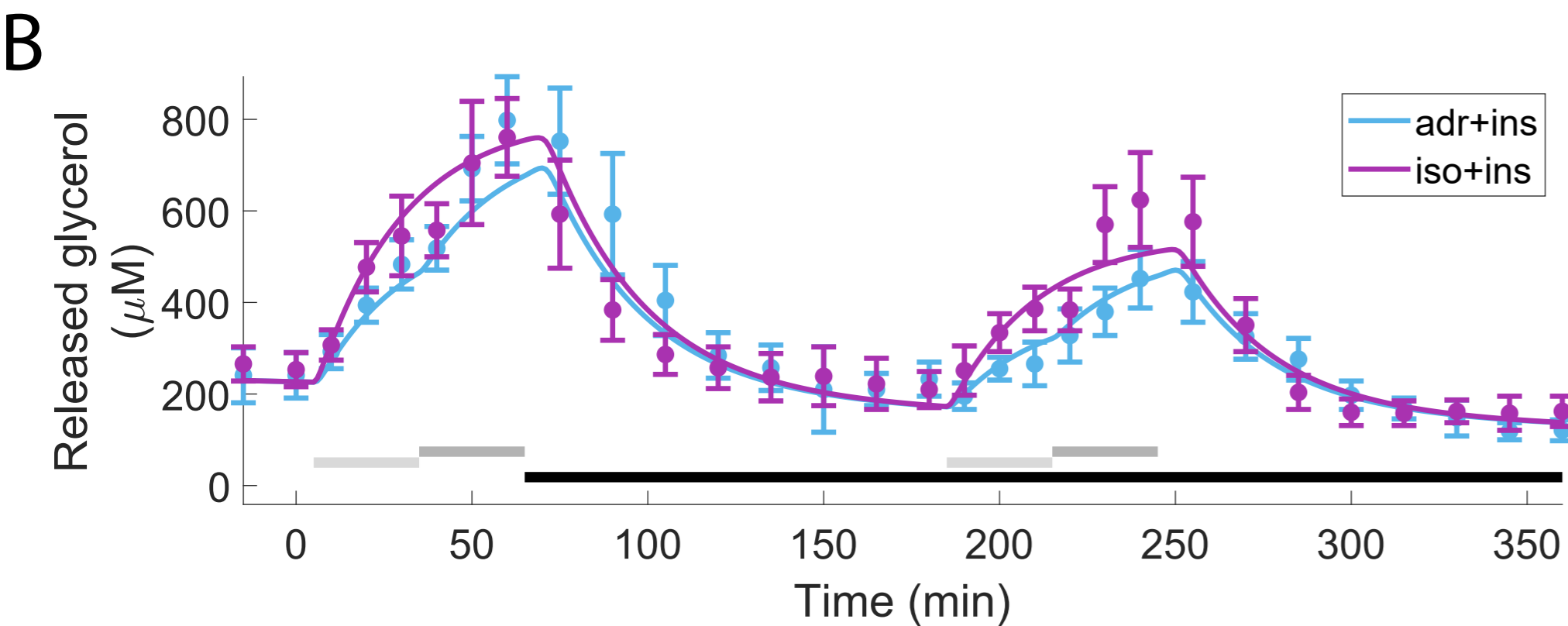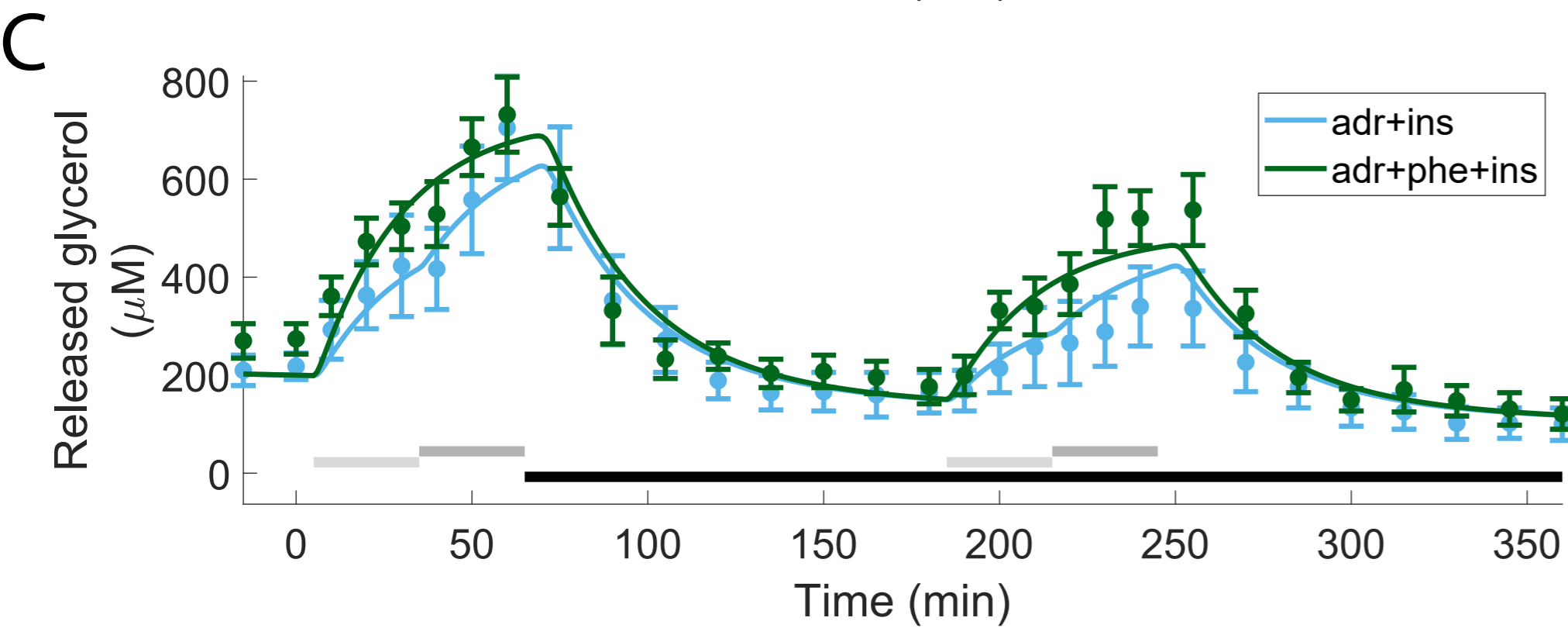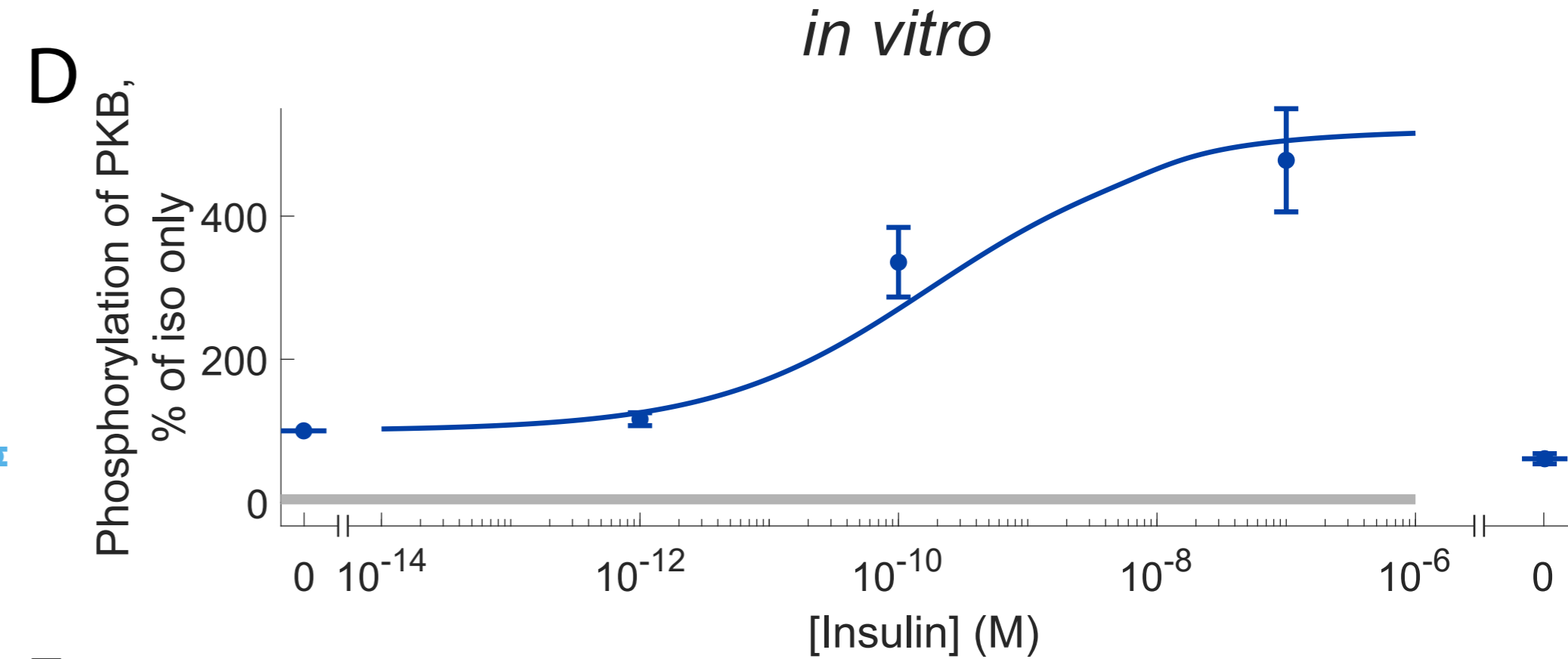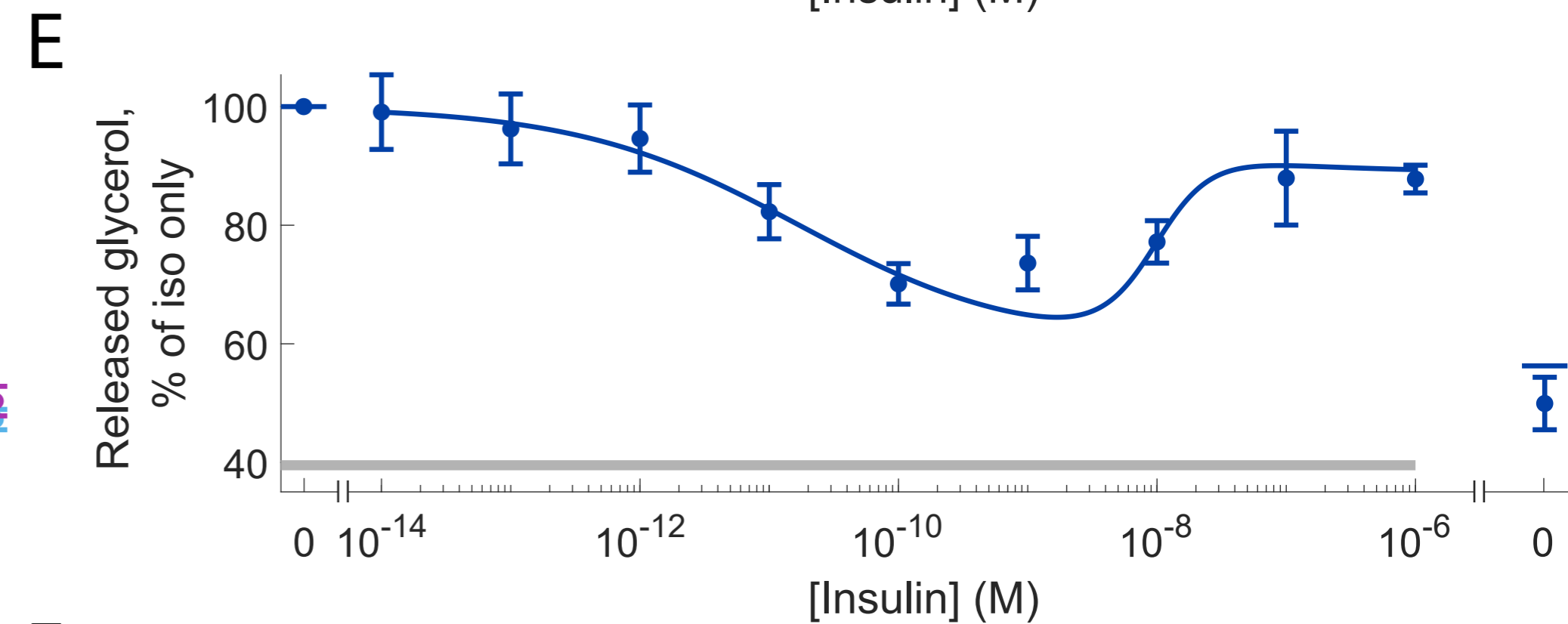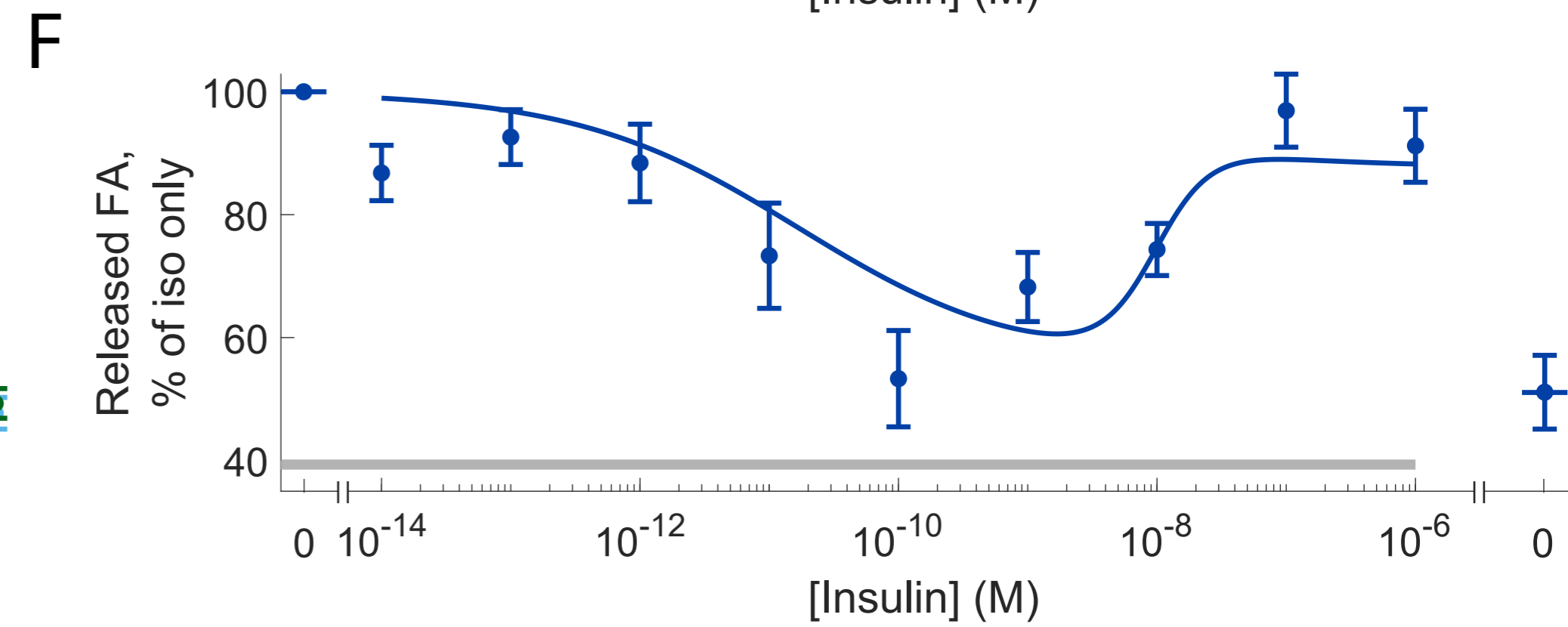

Supplement: S3 Fig — In all panels, solid lines represent the model simulation with the best agreement to data, the shaded areas represent the model uncertainty, and experimental data point are represented as mean values with error bars (SEM). (A-C), in vivo time-series experiments. (D-F), the in vitro dose-response experiments. In all subfigures, horizontal bars indicate where stimulations were given. In (A-C), light/dark grey bars indicate low/high adrenergic stimulus (1/10 μM adrenaline or 0.1/1 μM isoproterenol) with or without phentolamine (phe; 100 μM), black bars indicate stimulation with insulin (0.6 nM). In (D-F) grey bars indicate stimulation with isoproterenol (10 nM). In the in vivo experiments, experiments with adrenaline are shown in light blue (A-C), with isoproterenol in purple (B), and with the combined stimulation with adrenaline and phentolamine in green (C). In the in vitro experiments (D-F), increasing doses of insulin were given together with 10nM isoproterenol in all points except one. The point without isoproterenol got no stimulus and is shown to the right in the graphs. (PDF) [file pone.0261681.s005.pdf]

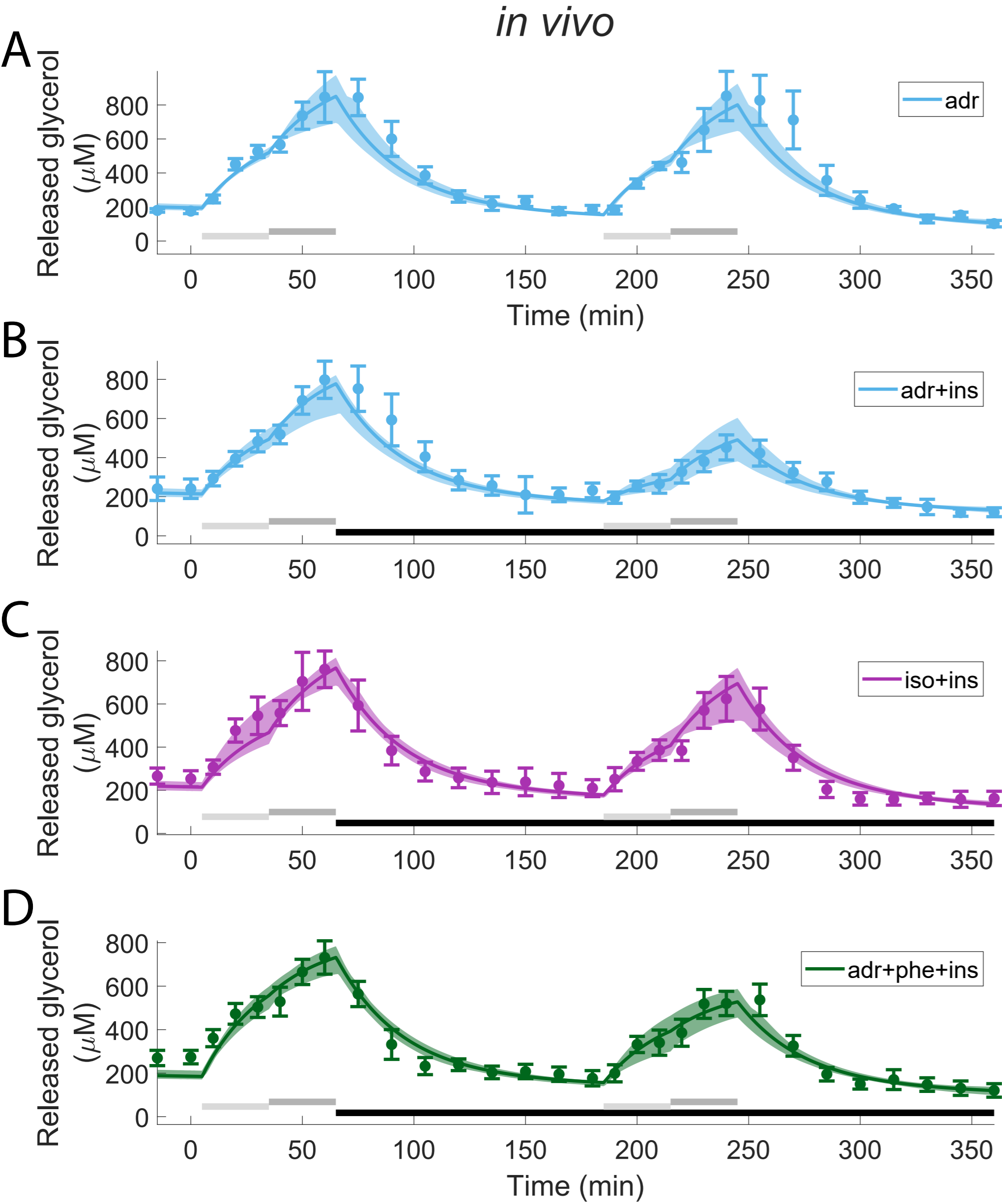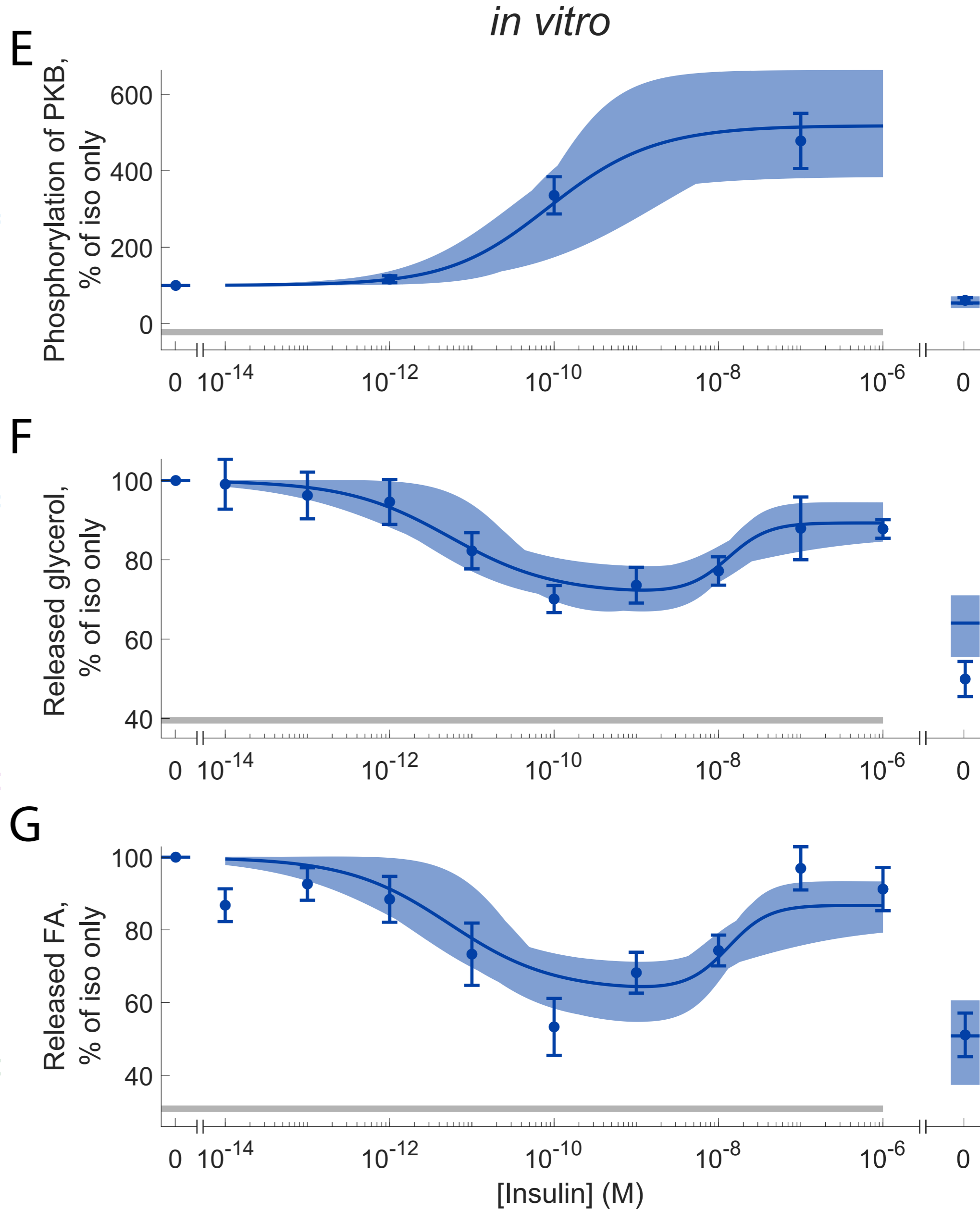

Supplement: S4 Fig — Estimation data and model simulations, for the original data (e.g. used in Fig 3). In all panels, solid lines represent the model simulation with the best agreement to data, and experimental data point are represented as mean values with error bars (SEM). (A-D), in vivo time-series experiments. (E-G), in vitro dose-response experiments. In all subfigures, horizontal bars indicate where stimulations were given. In detail, light/dark grey bars indicate stimulation with: 1/10 μM adrenaline in (A,B), 0.1/1 μM isoproterenol in (C), and 1/10 μM adrenaline with 100 μM phentolamine. Black bars in (B-D) indicates stimulation with 0.6 nM insulin. In (E-G) grey bars indicate stimulation with isoproterenol (10 nM). In the in vivo experiments, experiments with adrenaline are shown in light blue (A-C), with isoproterenol in purple (B), and with the combined stimulation with adrenaline and phentolamine in green (C). In the in vitro experiments (D-F), increasing doses of insulin were given together with 10 nM isoproterenol in all points except one. The point without isoproterenol got no stimulus and is shown to the right in the graphs. An alternative visualization is available in S5 Fig showing the difference by overlaying the experiments. (PDF) [file pone.0261681.s006.pdf]

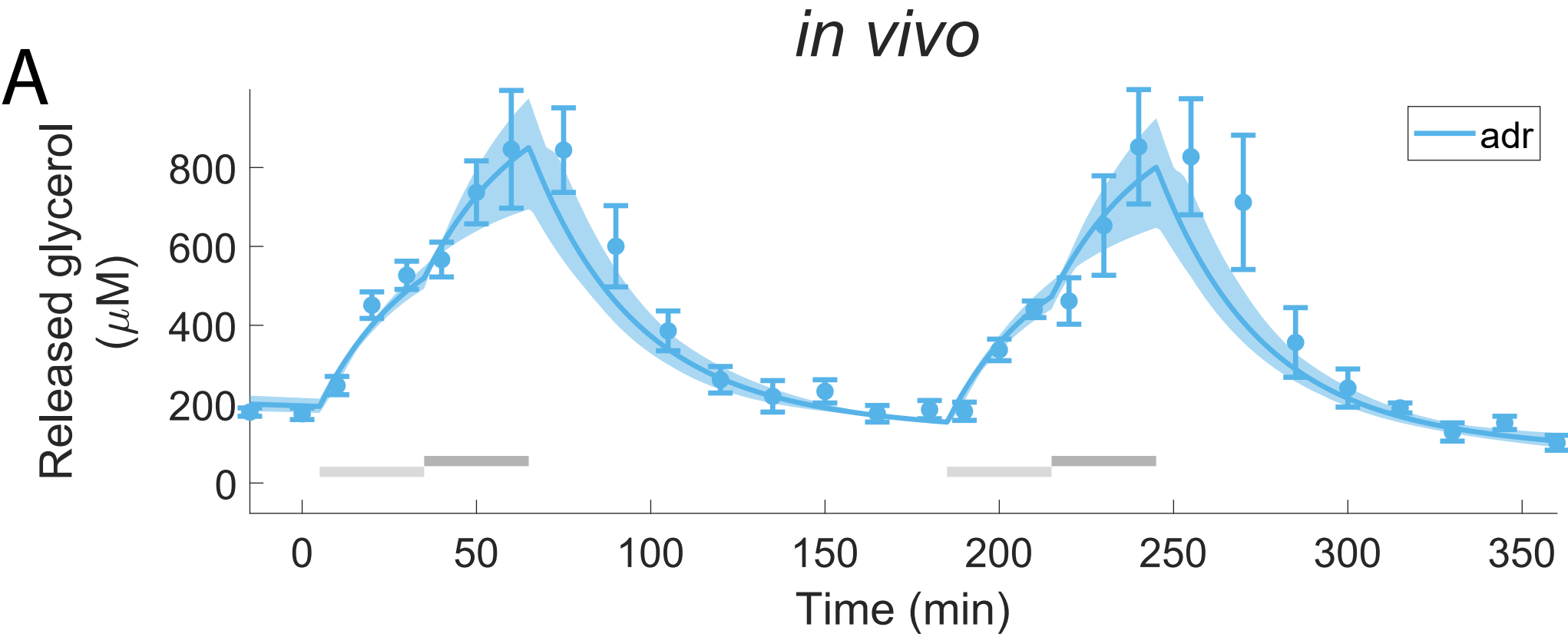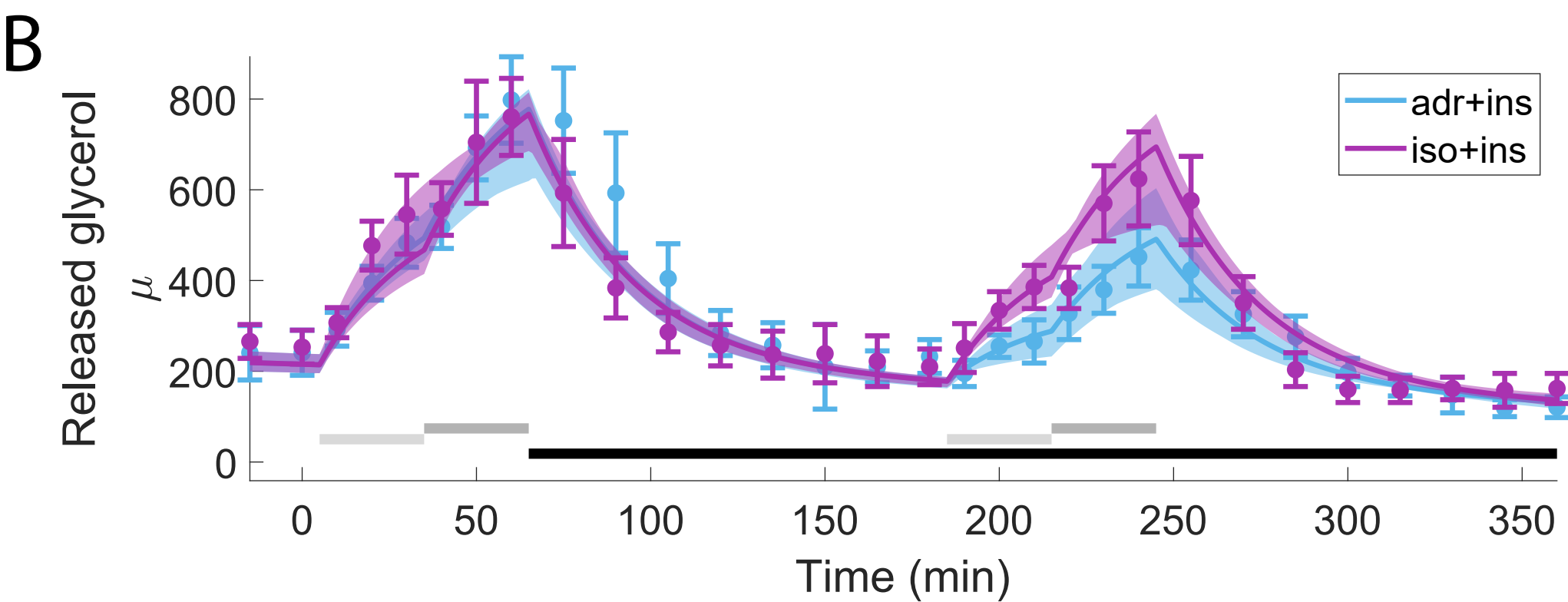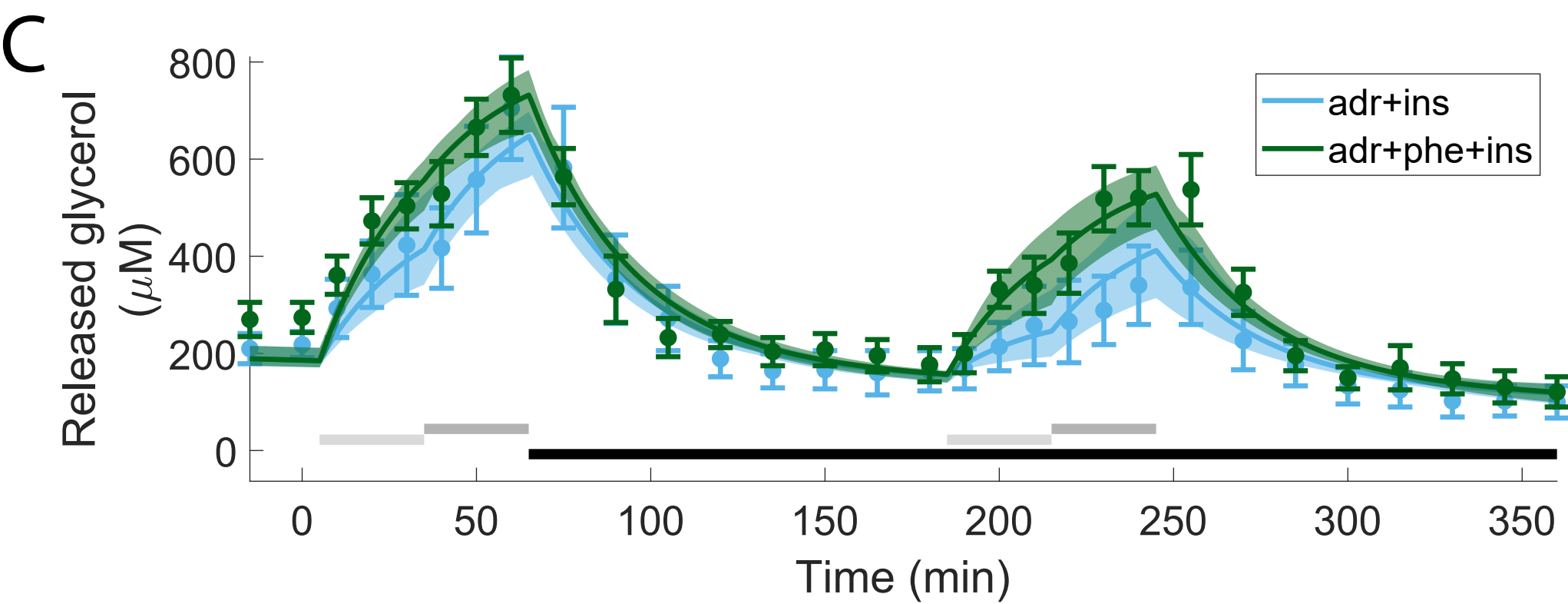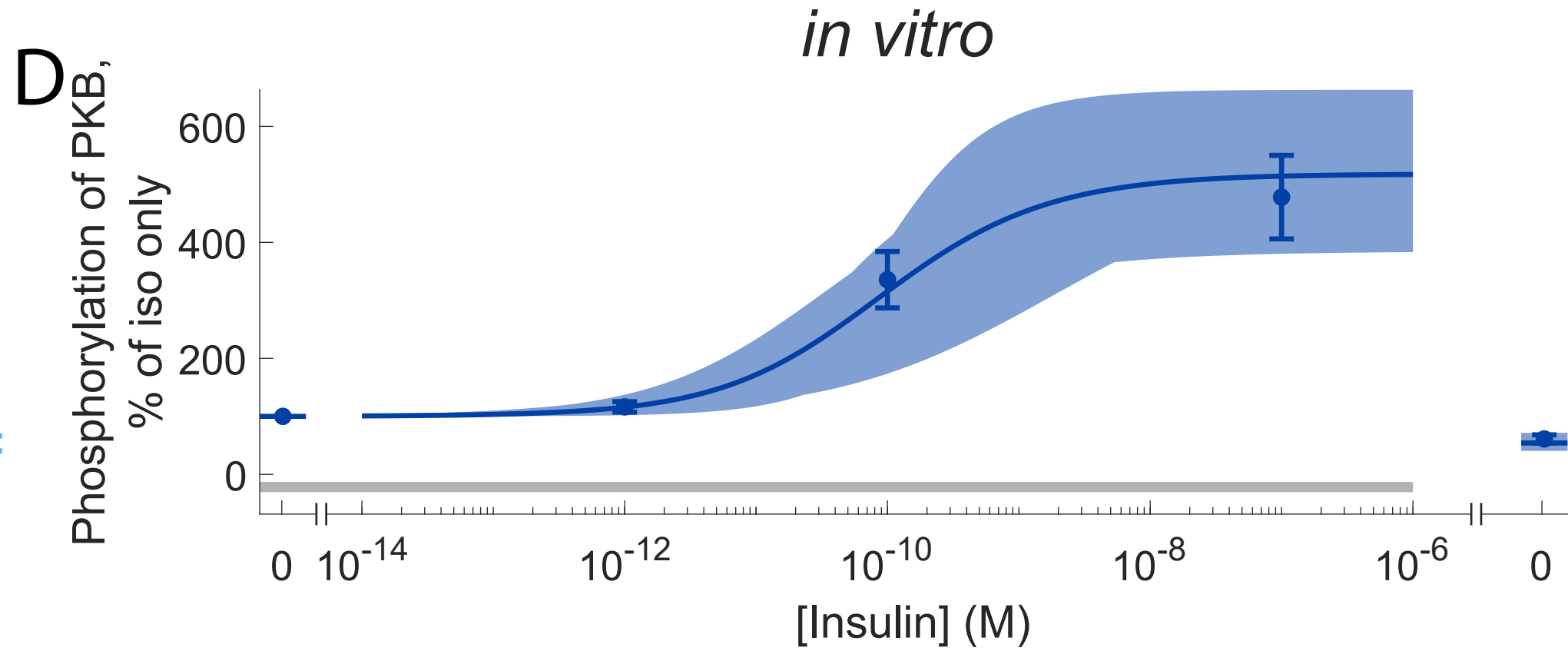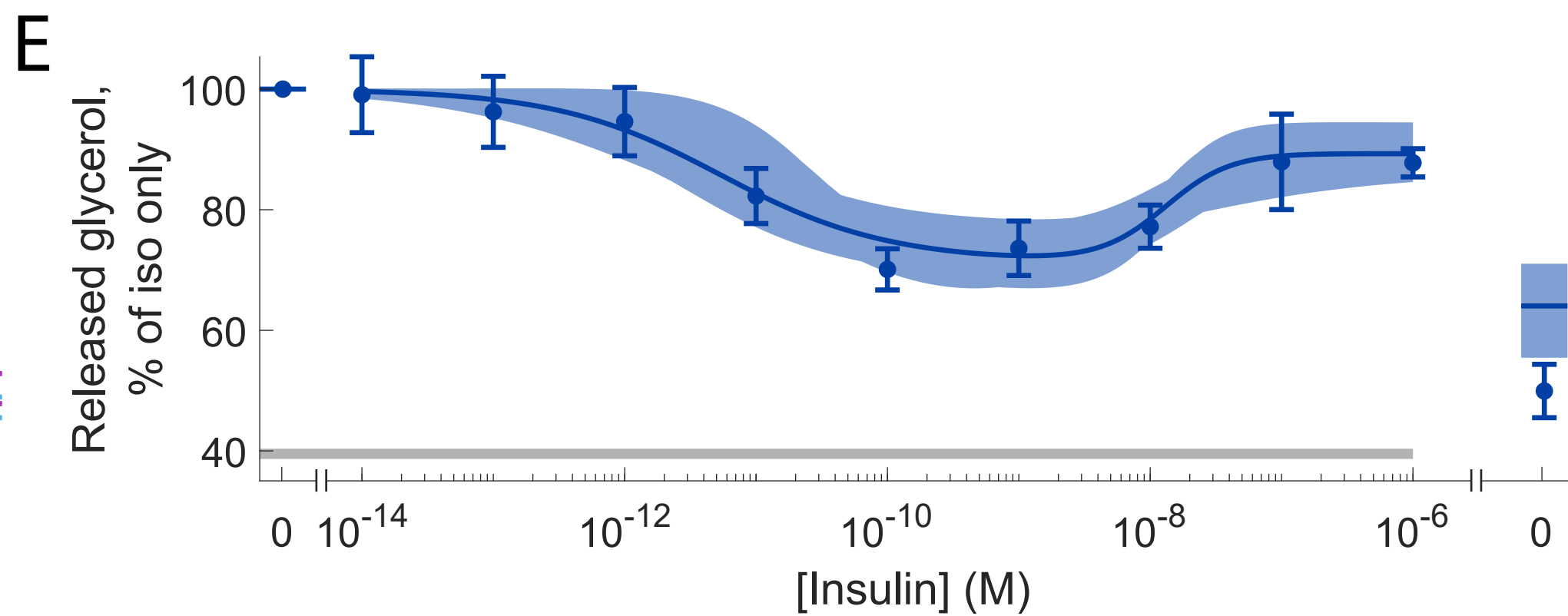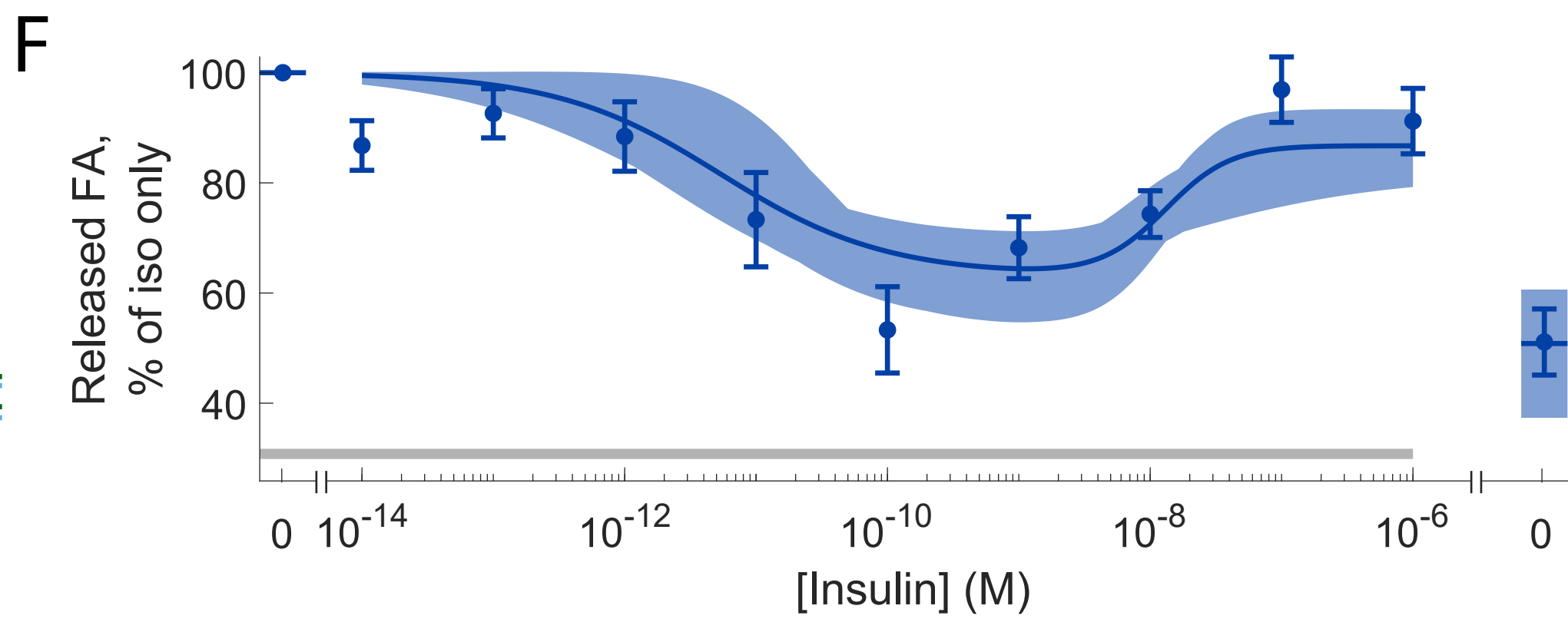

Supplement: S5 Fig — Estimation data and model simulations, for the original data (e.g. used in Fig 3). In all panels, solid lines represent the model simulation with the best agreement to data, the shaded areas represent the model uncertainty, and experimental data point are represented as mean values with error bars (SEM). (A-C), in vivo time-series experiments. (D-F), in vitro dose-response experiments. In all subfigures, horizontal bars indicate where stimulations were given. In (A-C), light/dark grey bars indicate low/high adrenergic stimulus (1/10 μM adrenaline or 0.1/1 μM isoproterenol) with or without phentolamine (phe; 100 μM), black bars indicate stimulation with insulin (0.6 nM). In (D-F) grey bars indicate stimulation with isoproterenol (10 nM). In the in vivo experiments, experiments with adrenaline are shown in light blue (A-C), with isoproterenol in purple (B), and with the combined stimulation with adrenaline and phentolamine in green (C). In the in vitro experiments (D-F), increasing doses of insulin were given together with 10nM isoproterenol in all points except one. The point without isoproterenol got no stimulus and is shown to the right in the graphs. (PDF) [file pone.0261681.s007.pdf]
