# Supplementary material for: A systems biology analysis of lipolysis and fatty acid release from adipocytes in vitro and from adipose tissue in vivo
Source: PLoS One. 2021 Dec 31;16(12):e0261681. doi: 10.1371/journal.pone.0261681 (PMC8719686; doi:10.1371/journal.pone.0261681)
Supplement: S1 Appendix — (PDF) [file pone.0261681.s008.pdf]

Appendix to "A systems biology analysis of lipolysis and fatty acid release from adipocytes *in vitro* and from adipose tissue *in vivo*"

William Lövfors[1,2], Jona Ekström[1], Cecilia Jönsson[3], Peter Strålfors[3], Gunnar Cedersund[1,4], Elin Nyman[1*]

1 Department of Biomedical Engineering, Linköping University, Linköping, Sweden
2 Department of Mathematics, Linköping University, Linköping, Sweden
3 Department of Biomedical and Clinical Sciences, Linköping University, Linköping, Sweden
4 Center for Medical Image Science and Visualization (CMIV), Linköping University, Linköping, Sweden

* Corresponding author: elin.nyman@liu.se, +46 13 286712, Department of Biomedical Engineering, Linköping University, 58183 Linköping, Sweden

1 Results from parameter estimating with the alternative dataset for *in vivo* glycerol release when stimulated with a combination of adrenaline and insulin

In the *in vivo* experiments, one dataset was repeated: the stimulation with a combination of adrenaline and insulin. We used the dataset resulting in the highest cost to get the most robust rejections, since having a too low cost could result in not rejecting a false model. Here, we have in short reproduced the results from the main paper, but using the alternative dataset. For simplicity, we refer to the dataset used in the main paper as the *main* dataset, and the alternative dataset used here as the *alternative* dataset. Since we are only testing if the conclusions drawn will be the same, we have not gathered the model uncertainties, only the optimal parameter sets.

2 Parameter identifiability

With regards to the parameter identifiability, we can draw essentially the same conclusions as in the main paper. All kinetic parameters are (downwards) identifiable, with the exception of $k8c$. For the discussion around $k8c$ we refer the reader to the section **Experimental observations and model development** in the main paper. As with the main dataset, we found 23 parameters to be identifiable. The parameter uncertainty bounds for the final minimal model is shown in Fig. 1 and Table 1.

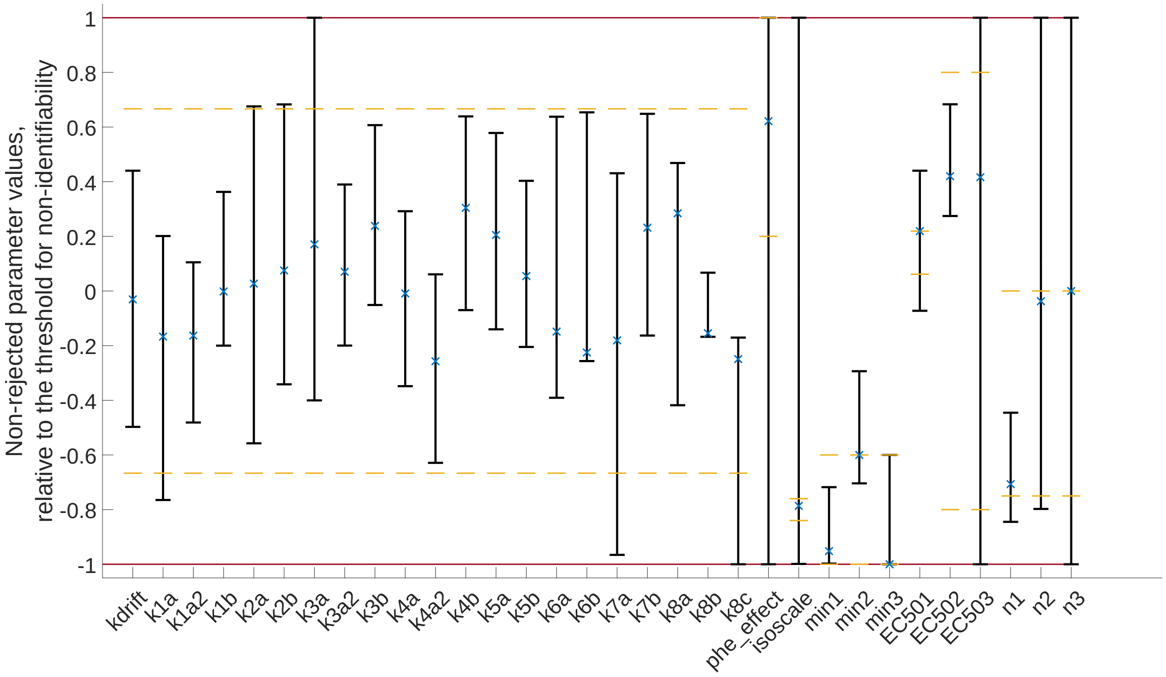

Figure 1: **Parameter identifiability analysis**. Comparable to Fig. 2 in the main paper. The minimal and maximal values of a parameter was found using the optimization approach detailed in the **Methods - Uncertainty estimation** section. The parameter values are expressed as relative values with respect to threshold for non-identifiability, where -1 represents a parameter value at the lower threshold, and 1 represents a parameter at the upper threshold. x represent the optimal parameter values and yellow dashed lines represent the bounds for a specific parameter when estimating parameter values. Bounds, thresholds and parameter values are shown in Table 1 and Table 2

Parameter	Lower threshold	Upper threshold	$\theta^{min}_{alternative}$	$\theta^{max}_{alternative}$
$kdrift$	10^{-9}	10^{9}	$3.3463 \cdot 10^{-5}$	$9.1549 \cdot 10^{3}$
$k1a$	10^{-9}	10^{9}	$1.3075 \cdot 10^{-7}$	$6.4646 \cdot 10^{1}$
$k1a2$	10^{-9}	10^{9}	$4.6632 \cdot 10^{-5}$	8.8201
$k1b$	10^{-9}	10^{9}	$1.5857 \cdot 10^{-2}$	$1.8380 \cdot 10^{3}$
$k2a$	10^{-9}	10^{9}	$9.6332 \cdot 10^{-6}$	$1.2044 \cdot 10^{6}$
$k2b$	10^{-9}	10^{9}	$8.4484 \cdot 10^{-4}$	$1.3983 \cdot 10^{6}$
$k3a$	10^{-9}	10^{9}	$2.4968 \cdot 10^{-4}$	$1.0000 \cdot 10^{9}$
$k3a2$	10^{-9}	10^{9}	$1.5935 \cdot 10^{-2}$	$3.2159 \cdot 10^{3}$
$k3b$	10^{-9}	10^{9}	$3.4584 \cdot 10^{-1}$	$2.9025 \cdot 10^{5}$
$k4a$	10^{-9}	10^{9}	$7.3391 \cdot 10^{-4}$	$4.2419 \cdot 10^{2}$
$k4a2$	10^{-9}	10^{9}	$2.1891 \cdot 10^{-6}$	3.5392
$k4b$	10^{-9}	10^{9}	$2.3475 \cdot 10^{-1}$	$5.6350 \cdot 10^{5}$
$k5a$	10^{-9}	10^{9}	$5.4670 \cdot 10^{-2}$	$1.6026 \cdot 10^{5}$
$k5b$	10^{-9}	10^{9}	$1.4359 \cdot 10^{-2}$	$4.2477 \cdot 10^{3}$
$k6a$	10^{-9}	10^{9}	$3.0427 \cdot 10^{-4}$	$5.4919 \cdot 10^{5}$
$k6b$	10^{-9}	10^{9}	$4.9210 \cdot 10^{-3}$	$7.6606 \cdot 10^{5}$
$k7a$	10^{-9}	10^{9}	$2.0387 \cdot 10^{-9}$	$7.5539 \cdot 10^{3}$
$k7b$	10^{-9}	10^{9}	$3.4069 \cdot 10^{-2}$	$6.8423 \cdot 10^{5}$
$k8a$	10^{-9}	10^{9}	$1.7294 \cdot 10^{-4}$	$1.6436 \cdot 10^{4}$
$k8b$	10^{-9}	10^{9}	$3.0972 \cdot 10^{-2}$	4.0045
$k8c$	10^{-9}	10^{9}	$1.0000 \cdot 10^{-9}$	$2.9167 \cdot 10^{-2}$
phe_effect	0	1	0.0000	1.0000
$isoscale$	0	10^{2}	$6.1918 \cdot 10^{-2}$	$1.0000 \cdot 10^{2}$
min_1	0	10^{2}	$1.1948 \cdot 10^{-1}$	$1.4109 \cdot 10^{1}$
min_2	0	10^{2}	$1.4806 \cdot 10^{1}$	$3.5324 \cdot 10^{1}$
min_3	0	10^{2}	0.0000	$2.0000 \cdot 10^{1}$
$EC50_1$	10^{-6}	10^{4}	$4.3451 \cdot 10^{-2}$	$1.5882 \cdot 10^{1}$
$EC50_2$	10^{-6}	10^{4}	2.3564	$2.6082 \cdot 10^{2}$
$EC50_3$	10^{-6}	10^{4}	$1.0000 \cdot 10^{-6}$	$1.0000 \cdot 10^{4}$
n_1	0	4	$3.1044 \cdot 10^{-1}$	1.1091
n_2	0	4	$4.0495 \cdot 10^{-1}$	4.0000
n_3	0	4	0.0000	4.0000

Table 1: **All bounds and estimated values for the free parameters**. The parameters were allowed to vary in the range given in S2 Table. For the specific parameter being investigated, the bound was relaxed and the threshold for when a parameter was deemed non-identifiable was set to the value given in the table in columns Lower threshold and Upper threshold). The minimum and maximal found values of a parameter is given in columns $\theta^{min}_{alternative}$, and $\theta^{max}_{alternative}$ respectively.

Parameter	Lower bound	Upper bound	$\theta^*_{alternative}$
$kdrift$	10^{-6}	10^{6}	$5.2879 \cdot 10^{-1}$
$k1a$	10^{-6}	10^{6}	$3.1457 \cdot 10^{-2}$
$k1a2$	10^{-6}	10^{6}	$3.3985 \cdot 10^{-2}$
$k1b$	10^{-6}	10^{6}	$9.6483 \cdot 10^{-1}$
$k2a$	10^{-6}	10^{6}	1.7521
$k2b$	10^{-6}	10^{6}	4.7452
$k3a$	10^{-6}	10^{6}	$3.4389 \cdot 10^{1}$
$k3a2$	10^{-6}	10^{6}	4.3051
$k3b$	10^{-6}	10^{6}	$1.3999 \cdot 10^{2}$
$k4a$	10^{-6}	10^{6}	$8.3017 \cdot 10^{-1}$
$k4a2$	10^{-6}	10^{6}	$4.8559 \cdot 10^{-3}$
$k4b$	10^{-6}	10^{6}	$5.4927 \cdot 10^{2}$
$k5a$	10^{-6}	10^{6}	$7.0210 \cdot 10^{1}$
$k5b$	10^{-6}	10^{6}	3.1047
$k6a$	10^{-6}	10^{6}	$4.5970 \cdot 10^{-2}$
$k6b$	10^{-6}	10^{6}	$9.4469 \cdot 10^{-3}$
$k7a$	10^{-6}	10^{6}	$2.3655 \cdot 10^{-2}$
$k7b$	10^{-6}	10^{6}	$1.2152 \cdot 10^{2}$
$k8a$	10^{-6}	10^{6}	$3.6163 \cdot 10^{2}$
$k8b$	10^{-6}	10^{6}	$4.0657 \cdot 10^{-2}$
$k8c$	10^{-6}	10^{6}	$5.7212 \cdot 10^{-3}$
phe_effect	0.6	1	$8.1080 \cdot 10^{-1}$
$isoscale$	8	$1.2 \cdot 10^{1}$	$1.0683 \cdot 10^{1}$
min_1	0	$2 \cdot 10^{1}$	2.4097
min_2	0	$2 \cdot 10^{1}$	$2.0000 \cdot 10^{1}$
min_3	0	$2 \cdot 10^{1}$	0.0000
$EC50_1$ (nM)	0.5	1.1	1.2454
$EC50_2$ (nM)	10^{-5}	10^{3}	$1.2605 \cdot 10^{1}$
$EC50_3$ (nM)	10^{-5}	10^{3}	$1.2080 \cdot 10^{1}$
n_1	0.5	2	$5.8643 \cdot 10^{-1}$
n_2	0.5	2	1.9252
n_3	0.5	2	2.0000

Table 2: **Bounds used for optimization of the free parameters, and the set of optimal values**. The rate parameters (kx) were given a free range (10^{-6} to 10^{6}). $isoscale$ was allowed a 20% deviation from the expected value of 10. For the input functions, the minimum values min_x was given a range from zero to 20% of max, the steepness n_x was given a range from 0 to 2, and the $EC50_x$ was given a free range for all doses used in the dataset from [15] (10^{-5} to 10^{3} nM), except for $EC50_1$ which was limited based on the EC50 of IR in [17]. $\theta^*_{alternative}$ corresponds to the optimal parameter set when using the alternative dataset.

3 Best agreement between model and data

The best model agreement to data are essentially the same when using the alternative dataset as with the main one. The best cost (v^*) is slightly lower when using the alternative dataset ($v^*_{alternative}$) compared to when using the main (v^*_{main}), and thus still below the threshold of rejection ($v^*_{alternative} = 128.2 < v^*_{main} = 130.8 < \chi^2(0.05, 137) = 165.3$). The model agreement for the best parameter set is shown in Fig. 2, and the values are given in Table 2.

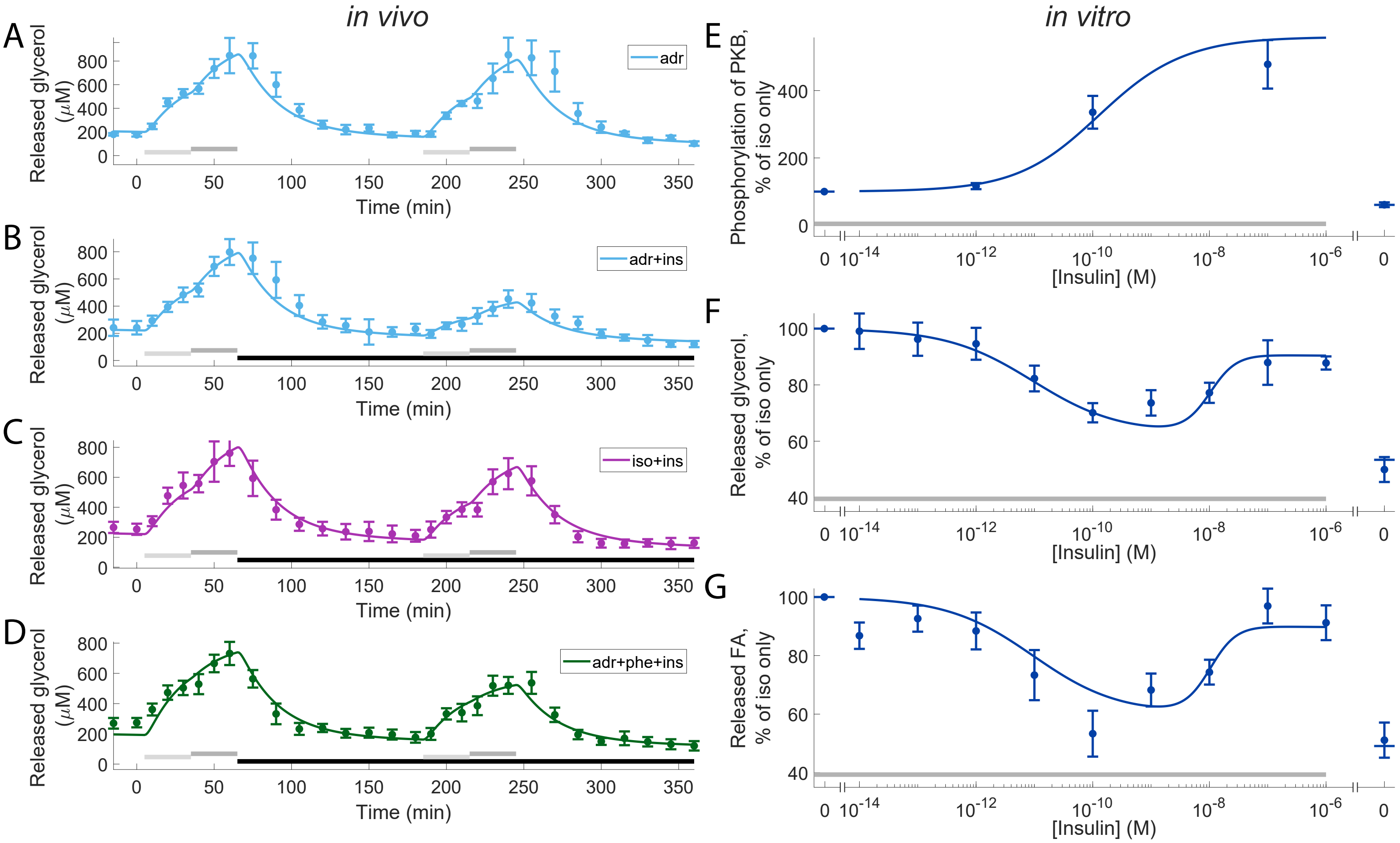

Figure 2: **Model agreement with experimental data**. In all panels, solid lines represent the model simulation with the best agreement to data, and experimental data points are represented as mean values with error bars (SEM). (A-D), *in vivo* time-series experiments. (E-G),*in vitro* dose-response experiments. In all subfigures, horizontal bars indicate where stimulations were given. In detail, light/dark grey bars indicate stimulation with: 1/10 µM, respectively, adrenaline in (A,B), 0.1/1 µM isoproterenol in (C), and 1/10 µM adrenaline with 100 µM phentolamine. Black bars in (B-D) indicates stimulation with 0.6 nM insulin. In (E-G) grey bars indicate stimulation with isoproterenol (10 nM). In the *in vivo* experiments, experiments with adrenaline are shown in light blue (A-C), with isoproterenol in purple (B), and with the combined stimulation with adrenaline and phentolamine in green (C). In the *in vitro* experiments (D-F), increasing doses of insulin were given together with 10 nM isoproterenol in all points except one.

4 Predicting HSL phosphorylation

The best model prediction of the HSL phosphorylation data could be slightly better when using the alternative data ($v^*_{HSL,alternative} 6.5 =< v^*_{HSL,main} = 10.7$). Thus, the model can still predict independent validation data when using alternative dataset.

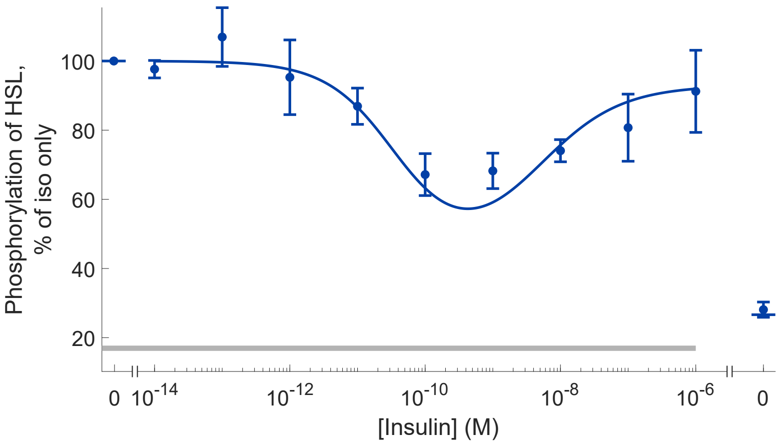

Figure 3: **Prediction of intracellular extent of HSL phosphorylation**. The line represent the model prediction with the best agreement with the validation data, and the experimental data are represented as mean values with error bars (SEM). The horizontal grey bar indicates where stimulation with isoproterenol (10 nM) have been given. Increasing doses of insulin were given together with 10 nM isoproterenol in all points except one. The point without isoproterenol got no stimulus and is shown to the right in the graph.

5 Removing insulin actions

In all cases, the optimal cost is slightly lower when using the alternative dataset ($v^*_{alternative}$) compared to the cost when using the main dataset (v^*_{main}). However, we draw the same conclusions when using the alternative dataset instead of the main dataset. In essence, removing insulin action-1 or -2 renders the model unable to agree with the data sufficiently well: $v^*_{alternative,1} = 496.9 < v^*_{main,1} = 501.1 > \chi^2(0.05, 137) = 165.3$ and $v^*_{alternative,2} = 179.6 < v^*_{main,2} = 181.8 > \chi^2(0.05, 137) = 165.3$ for the removal of action-1 and -2 respectively. Removing insulin action-3 yields a cost that is acceptable quantitatively ($v^*_{alternative,3} = 120.4 < v^*_{main,3} = 126.4 < \chi^2(0.05, 137) = 165.3$), but the agreement is qualitatively not good enough. The reason why the model is not qualitatively good enough without insulin action-3 is the same as for the main dataset used in the paper. In the data from the experiment without phentolamine (Fig. 4D, blue error bars) insulin inhibits the release of glycerol during the second set of stimuli with adrenaline (at 190 to 240 minutes) relative to the first set of stimuli with adrenaline without insulin stimulation (at 10 to 60 minutes). This inhibitory effect by insulin on the release of glycerol during the second set of stimulation with adrenaline is blunted in the data from the experiment with phentolamine (green error bars). Furthermore, the effect of phentolamine on the release of glycerol during the first set of stimuli with adrenaline is markedly lower than when insulin is added in the second set of stimuli. In other words, the effect of phentolamine on the release of glycerol is in a sense insulin dependent. This behaviour

of phentolamine having an insulin dependent effect is not exhibited by the model when insulin action-3 is removed. In Fig. 4D, the effect of adding phentolamine can seen by comparing the simulation with phentolamine (green line) with the simulation without phentolamine (blue line). The effect of phentolamine is essentially the same in both sets of stimuli with adrenaline. In other words, the effect of phentolamine is not insulin dependent in the model simulations. Therefore, the model without insulin action-3 is not qualitatively good enough. Consequently, all three actions of insulin are required for the model to explain the available experimental data.

The effect of removing either action from the model is the same when using the alternative dataset as when using the main one. Again, we refer the reader to the section **Investigating the different actions of insulin** in the main paper. The effects of removing either of the actions are shown in Fig. 4.

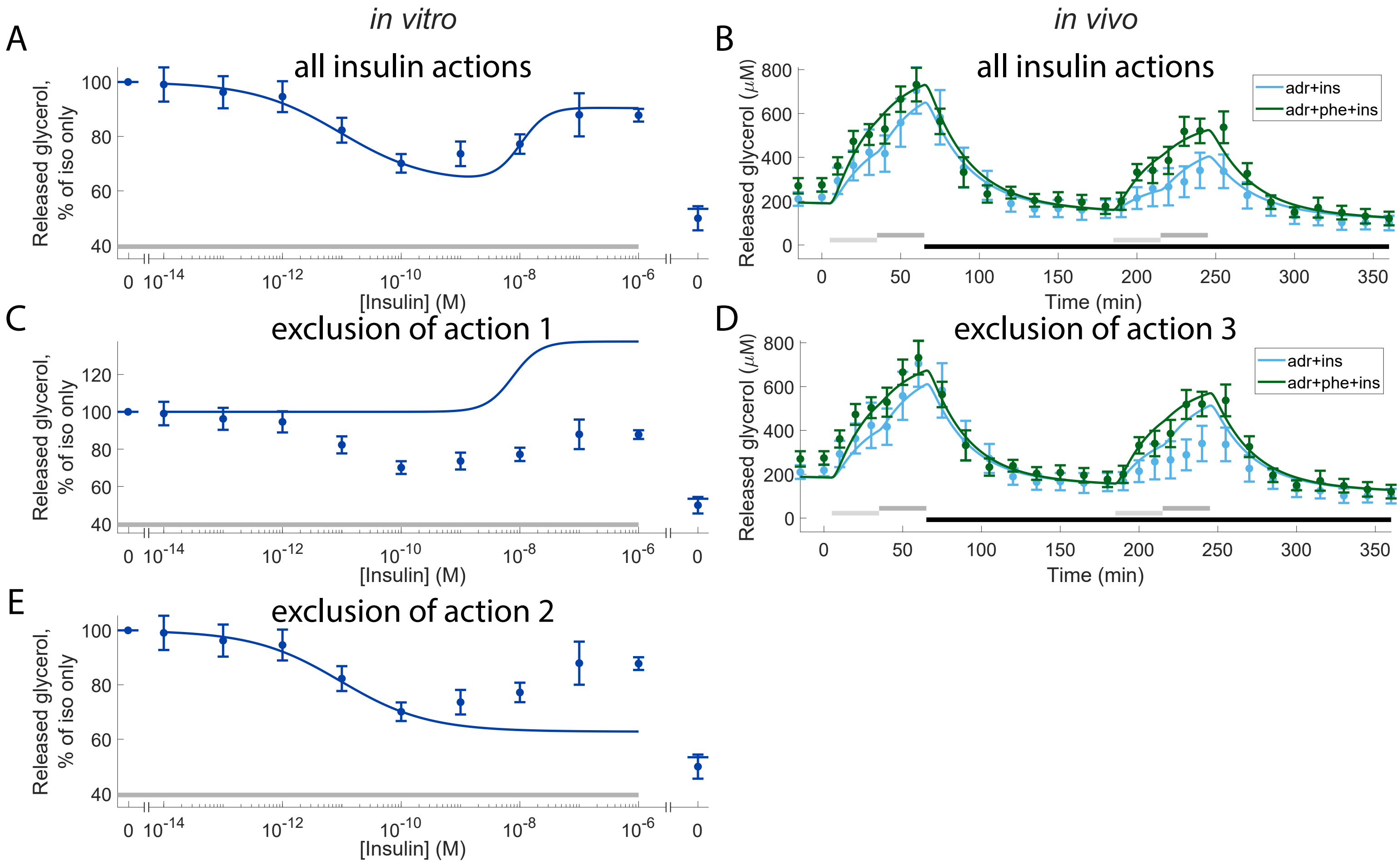

Figure 4: **Effects of excluding either of the three insulin actions**. In panels (A-E), data points with error bars represent mean and SEM values and solid lines represent the model simulation with the best agreement to data. (A, B), model simulations with all insulin actions present (same as Fig. 2C, F), see Fig. 1 in the main paper for a graphical representation of the three insulin actions. (C-E), the model simulations when either of the actions are excluded. In all subfigures, horizontal bars indicate where stimulations were given. In (A, C, E) grey bars indicate stimulation with isoproterenol (10 nM) and in (B, D) light/dark grey bars indicate stimulation with low/high dose of adrenaline (1/10 µM) with or without phentolamine (100 µM), and black bars indicate stimulation with insulin (0.6 nM). In the *in vitro* experiments (A, C, E), increasing doses of insulin were given together with 10nM isoproterenol in all points except one. The point without isoproterenol got no stimulus and is shown to the right in the graphs. In the *in vivo* experiments, experiments with adrenaline are shown in light blue (B, D) and with the combined stimulation with adrenaline and phentolamine in green (D).

6 Summary

In summary, the overall cost of the model with respect to the estimation data is slightly lower when using the alternative dataset. Thus the distance to the threshold of rejection is slightly wider, yielding slightly more freedom to the model when making prediction, and consequently slightly wider model predictions. However, no conclusions from the main paper is altered.
