# Supplementary material for: A systems biology analysis of lipolysis and fatty acid release from adipocytes in vitro and from adipose tissue in vivo
Source: PLoS One. 2021 Dec 31;16(12):e0261681. doi: 10.1371/journal.pone.0261681 (PMC8719686; doi:10.1371/journal.pone.0261681)
Supplement: Attachment — Submitted filename: Lovfors_etal.pdf [file pone.0261681.s009.pdf]


Referee's reviewing – *"A systems biology analysis of lipolysis and fatty acid release from adipocytes in vitro and from adipose tissue in vivo"*

Suggestions and comments to authors:

I have read this manuscript with great care and interest. The authors present a mathematical model that links *in vitro* and *in vivo* information on the action of insulin in the lipolysis mechanism of patients with type 2 diabetes mellitus. In particular, the work presented offers insight into the high circulation of fatty acids in patients under this pathological condition. The integrated model of *in vitro* and *in vivo* data provides an insight into the mechanisms of ectopic fat storage.

I consider the results to be scientifically interesting, mainly for a broad computational and/or theoretical biology community. The authors present a mathematical model of hormonal control of lipolysis in human adipose tissue, linking molecular events at the cellular level with corresponding responses at the tissue level. Although the procedures and results of the article are potentially suitable for a *PLOS ONE* journal; I must encourage the authors to make a thorough review of the submitted manuscript, taking carefully into account the following points.

1. The authors propose a mathematical model that links the dynamics of systems that belong to different length and time scales. On the one hand, there are the phenomena described at the tissue level and, on the other, those that are defined at the molecular level. The defined equations all contain the variable time, and there is no definition of the units specific to each scale. The time and characteristic length of the physical variables used must be defined in each scale. The equations themselves will reflect a crossover between the description at the molecular level and the description at the tissue level. This work would be greatly enhanced by adding this discussion to the original manuscript.

2. The proposed model is built under the conditions that generate three crosstalk mechanisms for the actions of insulin to explain the behavior observed in the experimental

data. However, the behavior of the data under the χ^2 statistician only ensures that the experimental phenomenology is recovered by suppressing the actions of insulin 1 and 2. This suggests that the frequency distribution that best describes the collected data is not necessarily of the type χ^2. Did the authors analyze other types of probability distributions to which their data could fit? Or, did they test any regression analysis to obtain models that support the inclusion of insulin actions 1 and 2? This analysis is inexpensive and could capture a greater number of features of the studied phenomenology.

3. The authors' hormonal control model of lipolysis includes insulin, adrenergic signaling, and an intracellular compartment. Although it is presumed to be the first model considering these aspects, it is still a minimal mathematical compartmental model. I appeal to the benefits of the mathematical model used for the authors to discuss the extent to which each of these factors influences reesterification in the diabetic condition, given that the model correctly captures the increased release of fatty acids *in vivo* in adipose tissue in patients with this condition and, even more, in conditions of insulin treatment. Again, the refinement of the minimum model used for modeling, plus the *in vivo* information, provides conditions for the authors to refine the model presented in the sense I indicated in the previous point. For example, in *Scientific reports*, **10**(1), 1-11, the authors suggest that the mathematical model presented there can be extended to the conditions you propose in this analysis. Confirming that these models can describe similar phenomenologies gives way to constructing a model of greater scope than those of the minimal type.

4. I suggest that the authors compose a vector space from the solution of the complete system of proposed differential equations. Under this mathematical structure, it is possible to tackle with greater rigor the problem of minimizing uncertainty and its independence with the initialization of the equations. Additionally, this algebraic structure would open a possibility to carry out principal component analysis that would locate and label the sources of error coming from your method. This could even optimize convergence times in numerical analyzes. This point is merely a suggestion

that, if explored, would yield an elegant and robust theory that would capture the processes of homeostasis, lipolysis, and hormonal adrenergic control in a single set of equations.

Further review:

While the research presented by the authors may be of sufficient interest to the theoretical biology or computational biology community, their work has sufficient potential to capture the attention of other scientific communities such as medicine, applied mathematics, and bioengineering, among others. This is due to the multi and interdisciplinarity latent in the manuscript they submit. For this, a slight restructuring of the wording presented would be sufficient. In particular, an introduction with a broader scope than that indicated, a new bibliography review that adds and updates some of its references, and a section with conclusions, perspectives, and limitations of the work, would undoubtedly raise the scientific quality of your research. In general terms, except for this point of structurality in the use of written scientific communication, his work is of the highest quality.

I hope, sincerely, that these comments are useful to the authors and are seen exclusively as constructive criticism for improving the already excellent work they present.
