# Supplementary material for: A systems biology analysis of lipolysis and fatty acid release from adipocytes in vitro and from adipose tissue in vivo
Source: PLoS One. 2021 Dec 31;16(12):e0261681. doi: 10.1371/journal.pone.0261681 (PMC8719686; doi:10.1371/journal.pone.0261681)
Supplement: Attachment — Submitted filename: Response to Reviewers.pdf [file pone.0261681.s010.pdf]

Response to reviewers

We kindly thank the reviewers for their time and effort in reviewing this paper. In this document we respond to the comments, with our response – written with blue text– where we believe a response to be of relevance.

Comments to the Author

Reviewer #1

Reviewer #1: In this study, Lövfors et al. developed a minimal mechanistic model to assess the impact of hormonal control of human adipocyte intracellular triglyceride hydrolysis (lipolysis). Several hormones regulate human adipocyte lipolysis: (Nor)epinephrine act via alpha2- and beta1/beta2-adrenergic receptors (AR) while the functional role of beta3-AR in humans is controversial; and insulin acts through the insulin receptor (IR). Between these different signaling pathways, a high degree of crosstalk exists that depends on hormone concentration. Thus, this new model shall enable to predict lipolysis during different metabolic conditions.

This is a very important topic as lipolysis is dysregulated in metabolic diseases such as obesity and type 2 diabetes and mathematic models will help to develop potential new pharmaceutical targets.

To feed their mathematic models, the authors have used available data from two different already published studies: one of their own publication (doi:10.1042/BCJ20190594) and one from Stich et al. (doi:10.1152/ajpendo.00502.2002). The data sets comprise in-vitro and in-vivo experiments using isolated mature adipocytes from subcutaneous adipose tissue and microdialysis data sets - both from non-diabetic individuals.

In vitro, lipolysis was stimulated for 10 min using 10 nM of the beta-AR agonist isoproterenol (ISO) and inhibited by varying insulin concentrations. In situ, lipolysis was stimulated by either ISO (0.1 and 1 µM) or epinephrine (1 and 10 µM) and inhibited by insulin or phentolamine (a nonselective alpha-AR antagonist) in 8 healthy subjects. Two cylces of repeated epinephrine perfusions had been used to feed the model instead of three that had been conducted in order to exclude possible effects of receptor desensitization. Fatty acid (FA) and glycerol released into the media and in the extracellular fluid were determined as a measure of intracellular lipolysis. In addition, immunoblot analyses of the phosphorylation status of two important proteins involved in intracellular lipolysis, namely hormone-sensitive lipase and protein kinase B, have been included in the in-vitro model. However, and this owes to the published data, information on other parameters that are crucial for lipolysis such as adipose triglyceride lipase, alpha/beta hydrolase domain containing protein 5, IR are missing.

The model included three different actions of the insulin: i) Inhibition of lipolysis via protein kinase B and phosphodiesterase 3B (PDE3B); ii) Stimulation of lipolysis via inhibition of PDE3B; and iii) Inhibition of lipolysis via alpha-AR. The experimental settings are comprehensible and have been previously published. Thus, the two studies are reasonable to be used for mathematic modeling.

My expertise does not lay in the field of mathematic modeling. Thus, I cannot comment on neither the modeling part nor the applied statistical tests. The authors have sufficiently referenced the literature and all findings are available in the main or supplemental text of the ms.

Overall, the manuscript is very well written in standard English without any obvious typos or

grammatical errors. The graphs are clearly presented and described. However, I suggest that the authors are more precise on the title that fits to the study.

We believe that Reviewer #1 has correctly understood our manuscript, and we are happy that the reviewer finds it to be well written. We have considered the suggestion to be more precise on the title of the manuscript, and we have not been able to find a more precise title.

Reviewer #2

Reviewer #2: I have read this manuscript with great care and interest. The authors present a mathematical model that links in vitro and in vivo information on the action of insulin in the lipolysis mechanism of patients with type 2 diabetes mellitus. In particular, the work presented offers insight into the high circulation of fatty acids in patients under this pathological condition. The integrated model of in vitro and in vivo data provides an insight into the mechanisms of ectopic fat storage.
I consider the results to be scientifically interesting, mainly for a broad computational and/or theoretical biology community. The authors present a mathematical model of hor- monal control of lipolysis in human adipose tissue, linking molecular events at the cellular level with corresponding responses at the tissue level. Although the procedures and results of the article are potentially suitable for a PLOS ONE journal; I must encourage the authors to make a thorough review of the submitted manuscript, taking carefully into account the following points.

Further review is attached as a pdf file.
We have copied the text from the pdf file and answered point-by-point below.

Referee's reviewing – *"A systems biology analysis of lipolysis and fatty acid release from adipocytes in vitro and from adipose tissue in vivo"*

Suggestions and comments to authors:

I have read this manuscript with great care and interest. The authors present a mathematical model that links *in vitro* and *in vivo* information on the action of insulin in the lipolysis mechanism of patients with type 2 diabetes mellitus. In particular, the work presented offers insight into the high circulation of fatty acids in patients under this pathological condition. The integrated model of *in vitro* and *in vivo* data provides an insight into the mechanisms of ectopic fat storage.

I consider the results to be scientifically interesting, mainly for a broad computational and/or theoretical biology community. The authors present a mathematical model of hormonal control of lipolysis in human adipose tissue, linking molecular events at the cellular level with corresponding responses at the tissue level. Although the procedures and results of the article are potentially suitable for a *PLOS ONE* journal; I must encourage the authorsto

make a thorough review of the submitted manuscript, taking carefully into account the following points.

We believe that Reviewer #2 have correctly understood our manuscript, and we are thankful for the suggestions.

1. The authors propose a mathematical model that links the dynamics of systems that belong to different length and time scales. On the one hand, there are the phenomena described at the tissue level and, on the other, those that are defined at the molecular level. The defined equations all contain the variable time, and there is no definition of the units specific to each scale. The time and characteristic length of the physical variables used must be defined in each scale. The equations themselves will reflecta crossover between the description at the molecular level and the description at the tissue level. This work would be greatly enhanced by adding this discussion to the original manuscript.

All ODEs are expressed in the time-scale of minutes, insulin is expressed in nM for the insulin actions. We have also added this information to the manuscript, at the beginning of the **Mathematical** modelling section.

In a sense our model is a model of an average adipocyte, where the outputs (glycerol and fatty acids) are scaled to the tissue scale (in vivo). At the same time, all in vitro experimental data are expressed as fold changes. We do the same transformations in the simulations as in the data. In other words, we also scale the model simulations to be expressed as fold change during the comparison between the model and the in vitro data such that the simulations and data are expressed in the same way. Together this means that while the model can accurately explain the available biological data, the parts of the model where no biological data is available might be inaccurate. In a sense it is more relevant to see the model as having the right dynamics, and the work herein as a test of the structure of the model, rather than a fully detailed description of all states and scales. We have added a paragraph on this in the Discussion section (second to last paragraph) of the manuscript.

2. The proposed model is built under the conditions that generate three crosstalk mechanisms for the actions of insulin to explain the behavior observed in the experimental data. However, the behavior of the data under the χ^2 statistician only ensures thatthe experimental phenomenology is recovered by suppressing the actions of insulin 1 and 2. This suggests that the frequency distribution that best describes the collected data is not necessarily of the type χ^2. Did the authors analyze other types of prob- ability distributions to which their data could fit? Or, did they test any regression analysis to obtain models that support the inclusion of insulin actions 1 and 2? This analysis is inexpensive and could capture a greater number of features of the studied

phenomenology.

We did not look at any other methods of rejection beyond the χ^2-test for the insulin actions-1 and -2. The reason for this is twofold. Firstly, because the cost of the model when the actions are removed are far beyond the threshold of rejection. Secondly, because the removal of either of the actions qualitatively removes important behaviours from the model. As can be seen in Fig. 5C and D, removal of insulin action-2 or -3 renders the model unable to exhibit either the initial decline, or the subsequent recovery at high insulin concentrations, in the release of glycerol.

Furthermore, we wanted to construct a minimal model that is in line with the current understanding of the biological system. Adding even more features would of course make the model able to capture more of prior knowledge but would also increase the risk of overfitting the model, which in turn could lead to a worse predictive power of the model.

3. The authors' hormonal control model of lipolysis includes insulin, adrenergic signaling, and an intracellular compartment. Although it is presumed to be the first model considering these aspects, it is still a minimal mathematical compartmental model.I appeal to the benefits of the mathematical model used for the authors to discussthe extent to which each of these factors influences reesterification in the diabetic condition, given that the model correctly captures the increased release of fatty acids *in vivo* in adipose tissue in patients with this condition and, even more, in conditions of insulin treatment. Again, the refinement of the minimum model used for modeling, plus the *in vivo* information, provides conditions for the authors to refine the model presented in the sense I indicated in the previous point. For example, in *Scientific reports*, **10**(1), 1-11, the authors suggest that the mathematical model presented therecan be extended to the conditions you propose in this analysis. Confirming that these models can describe similar phenomenologies gives way to constructing a model of greater scope than those of the minimal type.

The effect of insulin and adrenergic stimulation on the extent of reesterification in the diabetic condition is shown in Fig. 6D. In short, the addition of adrenergic stimulation slightly lowers the reesterification. The single point without adrenergic stimulation (furthest to the right) shows a slightly higher reesterification in the diabetic condition (yellow). On the other hand, increasing doses of insulin appears to have a negligible effect on the extent of reesterification. The estimated extent of reesterification for the diabetic condition (Fig. 6D) is markedly lower than in the normal condition due to a higher overall release of fatty acids (Fig. 6C), while the release of glycerol does not change (Fig. 6B). Biologically, an increase in released fatty acids in the diabetic state can be seen as a way for adipocytes to get rid of excess fat to avoid an overload of fat.

We have added the paragraph above to the **Discussion** section of the manuscript (the third to last paragraph).

While we agree that it would be of interest to eventually construct a more complex model that can both describe the processes in greater detail as well as to describe other (not yet included) features (such as other proteins). The aim of this work has been to construct a minimal model that can explain the available biological data. We therefore argue that it is beyond the scope of this paper to make a more complex model, but it is something we are planning to do in the future.

We were unfortunately unable to find the paper suggested, since the year and first author is lacking from the reference given.

4. I suggest that the authors compose a vector space from the solution of the complete system of proposed differential equations. Under this mathematical structure, it is possible to tackle with greater rigor the problem of minimizing uncertainty and its independence with the initialization of the equations. Additionally, this algebraic structure would open a possibility to carry out principal component analysis that would locate and label the sources of error coming from your method. This could even optimize convergence times in numerical analyzes. This point is merely a suggestion that, if explored, would yield an elegant and robust theory that would capture the processes of homeostasis, lipolysis, and hormonal adrenergic control in a single set of equations.

While we find the concepts raised by the reviewer to be interesting, we unfortunately also find them to fall a bit outside of our expertise and beyond the scope of this paper. However, we will explore these concepts in future work.

Further review:

While the research presented by the authors may be of sufficient interest to the theoretical biology or computational biology community, their work has sufficient potential to capture the attention of other scientific communities such as medicine, applied mathematics, and bioengineering, among others. This is due to the multi and interdisciplinarity latent in the manuscript they submit. For this, a slight restructuring of the wording presented would be sufficient. In particular, an introduction with a broader scope than that indicated, a new bibliography review that adds and updates some of its references, and a section with conclusions, perspectives, and limitations of the work, would undoubtedly raise the scientific quality of your research. In general terms, except for this point of structurality in the use of written scientific communication, his work is of the highest quality.

I hope, sincerely, that these comments are useful to the authors and are seen exclusively as

constructive criticism for improving the already excellent work they present.

Again, we thank the reviewer for all the constructive comments. We have tried to reformulate the introduction, to make it more appealing and interesting for a wider set of readers, including those proposed by the reviewer. We agree with the reviewer that it is important that topics like conclusions, limitations, and perspectives are important to include in a paper, and we have made sure that these topics are included in the Discussion. Regarding if these topics should have separate headings or not, we do understand that the reviewer would like to have a separate section for conclusions, perspective, and limitations, but we come from a tradition where these topics are discussed in the **Discussion** section. We hope that the reviewer agrees upon that we have covered these topics in the present discussion section.

Reviewer #3

Reviewer #3: The manuscript concerns a system biological analysis of a "minimal" mathematical model of the regulation and release of fatty acid via lipolysis. In general the peer is interesting and seems quite sound. However, even after reading all parts (partly several time)
there are still way too many aspect which remain unclear. The manuscript is clearly not ready for publication and should be carefully reworked before it could be suitable for publication.

In the following, I am only state some major examples and questions, which need to be addressed.

The process of data selection and parameter identification is unclear. For example that authors mention [4] as a source of measured data in the beginning, but not during the discussion of the Figs 3 ff. Moreover they only mention in the discussion why the data from [4] are reduced to the first two peaks. The proceedings leading to Tables S2 and S1 and their relationship is not made understandable. The author state that non-identifiable parameters are removed but not if these are parameters of the presented model or otherwise which processes have been eliminated from the model. They state that they brake their own rule by keeping parameter k8c, which should be discussed and argued better. The description of the uncertainty estimation is not clear, the property p hat not explained and it remains unclear in which sense it can be minimised. The authors do not address that removing insulin action 3 would pass their own chi^2 criteria of admissible parameters sufficiently. The model itself involves heuristic terms, sometimes nonlinear sometimes linear, a mixture which is sufficiently arbitrary, but can be imagined as a minimal model. However only one (not very good) example of data verification gives -- together with the above mentioned inconsistencies -- the feel that be model might be useful, but that its argumentation is weak.

We thank reviewer 3 for these detailed comments on things that should become more clear in the manuscript. We have addressed these concerns in the updated manuscript. More specifically, to clarify for the reader, we have now added a sentence to the start of the **Experimental observations and model development section**, to direct the reader to the **Data processing** section for details on how the data was processed. Here we state both the sources of the data, and how the data has been processed.

We now cite both sources of data when discussing the figures (third paragraph in the discussion).

Furthermore, we have also updated the section on parameter identifiability (third paragraph under **Experimental observations and model development**) to make it clear that no (downwards) non-

identifiable parameters are present in the model. We also updated the section to further clarify why the reesterification parameter k8c was not removed.

We have also elaborated on the uncertainty estimation in the **Methods – Uncertainty estimation** section and given examples on what properties the uncertainty can be estimated.

We have also extended the paragraph regarding the removal of insulin action-3, to further elaborate on why the insulin action-3 cannot be removed from the model.

Some more comments: Looking at the Fig. 4 and the parameters, one could speculate if the model for lipolysis is not good enough.
Basically the models assumes all hydrolysis steps saturated. Is PKBp equal PKB_A in Fig 1? The authors state that only one of the two data sets of [4] is used. What happens if the other one is used initially? The authors should comment on the 2/3 reesterification rate in healthy humans. Is it conceivable that evolution has produce a mechanism where 2/3/ of the produced FA are not released but immediately reesterfied?

Yes, PKB_P and PKB_A are the same. We have updated Fig. 1 from PKB_A to PKB_P.

We have updated Fig. 4 to now show the simulation with the best agreement to data, rather than the full uncertainty of the prediction, to make it clear that the model indeed accurately predict the data. We now instead present the full prediction with uncertainty in S4 Fig.

The two datasets are essentially the same, with the alternative dataset having a slightly larger uncertainty. Smaller uncertainties yield more robust rejections, and thus switching to the alternative dataset should not – and does not – impact the conclusions drawn in the paper. To confirm this, we reran the analysis with the alternative dataset, and found that the same conclusions should be drawn. We have added this analysis as a new supporting appendix (S7 Appendix).

We are now commenting on the reesterification in the Discussion:

It may appear surprising that evolution has provided us with the apparently wasteful reesterification of a substantial fraction of lipolytically released fatty acids. Nevertheless, the concept is far from new and is extensively discussed in e.g. [Jönsson 2019]. In effect, as fatty acids are fundamental to supply our energy requirements, they are also highly toxic and must always be strictly hindered from accumulating in cells. The reesterification is thus one aspect of the need to ensure that fatty acids do not accumulate, another aspect is the extremely intertwined regulatory pathways of lipolytic control
